# Riveted Interconnections of Capacitance‐Matched MXene‐Based Yarn Supercapacitors Enable Seamless Energy Integration in Textiles

**DOI:** 10.1002/smsc.202500229

**Published:** 2025-07-06

**Authors:** Neeraj Kumar, Patryk Wojciak, Shayan Seyedin

**Affiliations:** ^1^ School of Engineering Newcastle University Newcastle upon Tyne NE1 7RU UK

**Keywords:** asymmetric supercapacitors, energy storages, e‐textiles, graphene, MoS_2_, MXene, wearables

## Abstract

Electronic textiles are a transformative technology set to revolutionize next‐generation wearable devices. However, a major challenge is making efficient yarn‐based energy systems that power flexible wearables while blending seamlessly into textiles for unobstructed applications. Herein, 2D materials‐coated yarn supercapacitors (YSCs) are designed, offering a promising solution through capacitance‐matched electrode fabrication and a novel customizable riveted interconnection strategy for textile integration. MXene‐coated cotton yarns (negative electrode) achieve a remarkable specific capacitance of ≈7 360 mF cm^−2^ (≈536 F g^−1^). To complement the negative electrode, a positive yarn electrode (rGO/MoS_2_) is developed through a tailored synthesis process. A device fabrication strategy based on matching the capacitance of the yarn electrodes enhances the performance of YSCs, achieving an impressive specific capacitance of ≈658 mF cm^−2^ (≈53 F g^−1^), power density of ≈8,147 μW cm^−2^ (≈650 W kg^−1^), and energy density of ≈154.5 μWh cm^−2^ (≈12.3 Wh kg^−1^). The practical applicability of the YSCs is demonstrated via a novel yet simple integration design, whereby YSCs are connected to conductive rivets, which serve as buttons capable of toggling charge/discharge and easy removal from clothes for washing. The advancements made in this work enable on‐the‐go powering of wearable health systems, displays, and the Internet of things.

## Graphical abstract

High‐performance yarn supercapacitors (YSCs) using Ti_3_C_2_T_*x*_ MXene‐ and rGO/MoS_2_‐coated cotton yarns are developed for electronic textiles. Matching electrode capacitances maximizes voltage and energy density in asymmetric YSCs. An innovative riveted interconnection seamlessly integrates YSCs into an energy textile, enabling washability, improved aesthetics, and device protection. The energy textile powers small electronic devices, offering on‐the‐go energy solution for next‐generation soft wearables.

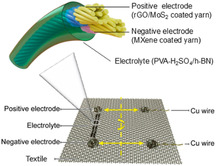

## 1 Introduction

Electronic textiles (E‐textiles) present a flexible, stretchable, and lightweight platform to enable new technological functionalities that extend beyond the limits of rigid and bulky electronics. Functional yarns with their 1D form factor once carefully integrated into garments enable the development of a new generation of soft E‐textiles that are capable of conforming to the contours of the human body. When used as devices, functional yarns can serve as building blocks of sensors, energy storage units, and a host of circuit components suitable for a plethora of applications, such as remote health and sports monitoring,^[^ ^1^ ^]^ virtual reality, real‐time data collection and analysis,^[^ ^2^ ^]^ computation,^[^ ^3^ ^]^ wireless communication,^[^ ^4^ ^]^ and display technologies.^[^ ^5^ ^]^ E‐textiles are proving to be a lucrative business venture, with the project global market growth from ≈$1.8 billion in 2025 to ≈$7.1 billion by 2035.^[^ ^6^ ^]^ A major contributor to this growth is the recent advancements in conductive yarns, which are key to integrating intelligent functionalities with otherwise passive textiles. As E‐textiles technology continues to advance, there is a growing need for energy storage solutions that match the inherent textile's flexible form factor and can be seamlessly integrated into garments to power the embedded electronic devices.^[^ ^7^ ^]^ Therefore, there is a high demand for next‐generation soft wearable yarns capable of storing energy, driving significant research efforts in this area.^[^ ^8^ ^]^ However, a significant challenge remains in seamlessly integrating yarn‐based energy storage systems into everyday textiles to enable their unobstructive application as flexible and high‐performance power supply units.

Among various energy storage devices, supercapacitors offer a suitable energy storage solution for E‐textiles applications, combining compactness, flexibility, rapid charging and discharging rates, long cycle life, enhanced safety, and cost‐effectiveness.^[^ ^9^ ^]^ Notably, supercapacitors have an advantage over the commonly used lithium‐ion batteries (LIBs) for E‐textiles applications because, unlike rigid and bulky LIBs, supercapacitors can be made flexible and yarn‐shaped and do not use flammable materials that can cause thermal runaway, representing a safer alternative to LIBs. For practical applications, flexible yarn‐based supercapacitors (YSCs) are required that demonstrate excellent mechanical flexibility and yarn form factor while also meeting the voltage and energy storage performance needed to power the electronic devices integrated into E‐textiles.^[^ ^10^ ^]^ YSCs typically consist of two yarn‐shaped electrodes separated by a solid or gel electrolyte, often in conjunction with a porous separator.^[^ ^11^ ^]^ The design of yarn‐shaped electrodes with high specific capacitances and their assembly into a suitable device structure that effectively utilizes the performance of each electrode are the most crucial steps in the development of high‐performance YSCs that are suitable for E‐textiles applications.

Recent studies have led to the development of various yarn‐shaped electrodes for YSCs.^[^ ^12^ ^]^ For instance, electrodes made of reduced graphene oxide (rGO)‐coated polyimide yarns were employed to develop YSCs that demonstrated a high specific capacitance of ≈1820 mF cm^−3^. In another study, Ni‐coated cotton yarns decorated with graphene were used as electrode to develop a solid‐state YSC, achieving a high specific capacitance of ≈110 mF cm^−1^.^[^ ^13^ ^]^ 2D materials have demonstrated an excellent potential as electrode materials for YSCs.^[^ ^8^, ^14^ ^]^ 2D MXenes (M_n+1_X_n_T_*x*_, M: transition metal, X: carbon and/or nitrogen, n: 1‐4, and T_*x*_: surface terminations), such as Ti_3_C_2_T_*x*_, have recently attracted widespread attention for supercapacitor applications, because of their exceptional electrical conductivity (up to ≈24 000 S cm^−1^),^[^ ^15^ ^]^ high specific capacitance (≈1,500 F cm^−3^),^[^ ^16^ ^]^ and high hydrophilicity (zeta potential of −30 to −60 mV in water).^[^ ^17^ ^]^ Combined with excellent mechanical properties (tensile strength of up to ≈670 MPa for ≈40 nm flakes^[^ ^18^ ^]^ and Young's modulus of ≈0.484 TPa),^[^ ^19^ ^]^ high skin compatibility, and nontoxicity,^[^ ^8^, ^20^ ^]^ MXenes also offer textile processability and safety for wearable applications. Ti_3_C_2_T_*x*_ MXene has indeed been used as an active material coating on various yarns, such as cellulose, nylon, and aramid (i.e., Kevlar), enabling the development of negative electrodes for YSCs due to the operation in the negative potential range.^[^ ^7^, ^8^ ^]^ For instance, a Ti_3_C_2_T_*x*_ MXene‐coated cellulose yarn electrode (≈77 wt% MXene loading) exhibited a specific capacitance of ≈759 mF cm^−1^ (at a scan rate of 2 mV s^−1^) within a potential range of −0.55 to 0.25 V.^[^ ^17^ ^]^

While the suitability of MXene‐coated yarns as negative electrodes has been demonstrated, research in YSCs has been mainly devoted to symmetric supercapacitor device design. Symmetric devices use the same positive and negative electrodes and offer limited voltage windows. For instance, Ti_3_C_2_T_*x*_ MXene was used to establish active material coating on both natural and synthetic yarns, enabling the fabrication of symmetric YSCs with a limited voltage window of 0.55 V, making them unsuitable for powering electronic devices.^[^ ^14^ ^]^ This is because even small electronic devices typically rated at 1.5 V and above. In contrast, asymmetric YSCs use different positive and negative electrodes and are preferred for E‐textiles applications due to their wider voltage ranges and higher energy densities compared to symmetric YSCs. To achieve optimal performance in asymmetric YSCs, it is essential to have matching capacitances for both electrodes, which is often challenging to achieve as electrode materials vary in terms of their energy storage performances. For instance, an asymmetric YSC was developed using a biscrolled Ti_3_C_2_T_*x*_ MXene/carbon nanotube (CNT) yarn as a negative electrode and a biscrolled RuO_2_/CNT electrode yarn as a positive electrode, enabling the cell voltage to be expanded up to 1.5 V, while demonstrating a specific capacitance of ≈554 mF cm^−2^ (at a current density of 2 mA cm^−2^).^[^ ^21^ ^]^ Matching the capacitance of the positive and negative electrodes offers a route to further enhance the overall capacitance of asymmetric YSCs. Importantly, the exceptionally high specific capacitance of MXene‐based electrodes has not been fully utilized for the development of high‐performance YSCs because of the lack of a suitable positive electrode that matches the capacitance of the negative MXene‐based electrode.

Hybridization of several 2D materials offers a potential route to designing a yarn‐based hybrid positive electrode with a suitable specific capacitance and operating potential range needed for the development of high‐performance YSCs that effectively utilize MXene‐based yarns as the negative electrode. For instance, Ti_3_C_2_T_*x*_ MXene/ rGO hybrid fibers with ≈88 wt% MXene loading were produced via a wet‐spinning process.^[^ ^22^ ^]^ These electrode fibers utilized both electrochemical double layer capacitance (EDLC) from rGO and pseudocapacitance from Ti_3_C_2_T_*x*_ MXene, demonstrating a high volumetric capacitance of ≈341 F cm^−3^ (at a current density of 0.5 A cm^−3^) while operating at a positive potential range of 0–0.8 V (vs. Ag/AgCl). Among the diverse range of 2D materials, semiconductor MoS_2_ could act as a pseudocapacitive electrode material offering a high specific capacitance (≈200 F g^−1^)^[^ ^23^ ^]^ and remarkable mechanical properties (tensile strength of ≈23 GPa and Young's modulus of ≈270 GPa).^[^ ^24^ ^]^ Additionally, MoS_2_ was integrated with EDLC materials, such as rGO, to develop a high‐performance hybrid electrode, achieving an exceptional specific capacitance (≈318 F g^−1^) within a positive potential range of 0–1.0 V (vs. Ag/AgCl).^[^ ^25^ ^]^ The rGO/MoS_2_ hybrid electrode offers a wide positive potential window combined with a synergistic effect, whereby, rGO offers EDLC and electrical conductivity and MoS_2_ provides an excellent pseudocapacitance.^[^ ^26^ ^]^ While rGO and MoS_2_ hybrids have been explored for supercapacitor applications,^[^ ^25^, ^27^ ^]^ their use as fibers or coated yarns for flexible positive electrodes in asymmetric YSCs remains unexplored. The design of a positive yarn electrode that matches the capacitance of Ti_3_C_2_T_*x*_ MXene negative yarn electrode requires carefully controlling the synthesis of rGO/MoS_2_ and its processing to achieve a desirable composition that maximizes specific capacitance.

Finally, YSCs will need to be integrated into textiles for practical applications as E‐textiles. Textile integration of YSCs must be seamlessly carried out using existing or compatible manufacturing processes that preserve the structural integrity and energy storage performance of YSCs while ensuring the textile host retains its inherent flexibility and breathability needed for wearable applications. Device interconnections are essential for linking multiple energy sources (to achieve suitable energy density and/or voltage) and for connecting the energy sources to various electronic components (e.g., display devices, energy harvesters, and sensors) embedded within a multifunctional E‐textile system. Interconnection strategies are well developed for strain/pressure sensors and heating fibers. However, a straightforward and reliable integration approach remains largely undeveloped for yarn‐based energy storage devices, highlighting a critical gap in the field. Notably, the development of an effective approach for the interconnection of YSCs to achieve series or parallel circuitry and establishing charging tabs have been the main challenges in the textile integration of YSCs, limiting their practical applications. Innovative approaches of textile integration using technologies that are compatible with both textiles and YSCs are critically required to accelerate the use of YSCs as practical energy storage solutions for E‐textiles.

**Figure 1:**
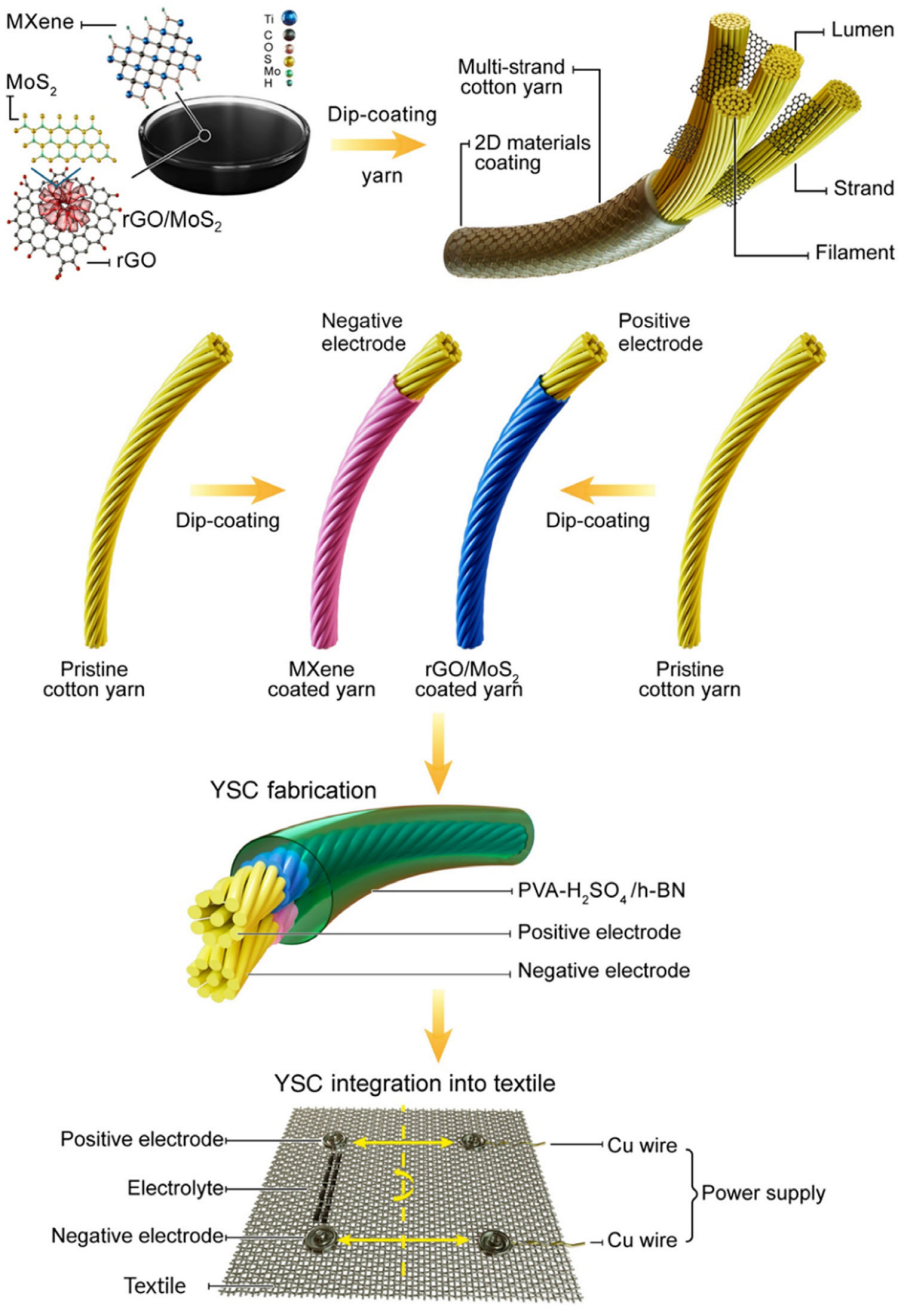
Schematic illustration of the dip‐coating process used in the development of cotton yarns coated with Ti_3_C_2_T_*x*_ MXene and rGO/MoS_2_ to form negative and positive electrodes, respectively, for YSCs fabrication, and integration into textiles using a snap rivet strategy.

In this work, we present a novel approach for the development of high‐performance and flexible asymmetric YSCs that effectively utilize the energy storage properties of the negative and positive electrodes achieved by coating Ti_3_C_2_T_*x*_ MXene and rGO/MoS_2_ on cotton yarns, respectively (**Figure** **1**). Cotton, composed mainly of natural and biodegradable cellulose polymer, offers abundant availability, suitable surface charges (−12.0 mV in neutral pH) to interact with the electrode materials,^[^ ^28^ ^]^ and excellent water absorption capacity (up to ≈24–26 times its mass).^[^ ^29^ ^]^ The Ti_3_C_2_T_*x*_ MXene‐coated yarns functioning as negative electrodes show enhanced energy storage capabilities indicative of their excellent aerial (gravimetric) capacitance of ≈7 360 mF cm^−2^ (≈536 F g^−1^) in the potential range of −0.6–0.2 V. We then develop capacitance‐matched asymmetric YSCs using a two‐step process consisting of 1) a hydrothermal synthesis of rGO/MoS_2_ that allows for a unique flower‐like morphology of MoS_2_ and varying the MoS_2_ loadings to maximize the energy storage performance of the positive electrode and 2) precisely matching the capacitance of the positive rGO/MoS_2_ yarn‐based electrode with that of the negative Ti_3_C_2_T_*x*_ MXene yarn‐based electrode by carefully adjusting the electrode lengths. The optimized rGO/MoS_2_‐coated yarns as positive electrodes show a high aerial (gravimetric) capacitance of ≈4,756 mF cm^−2^, (≈402 F g^−1^). Further, the YSCs are developed using a specifically tailored layer of polyvinyl alcohol (PVA)–sulfuric acid (H_2_SO_4_)/hexagonal boron nitride nanosheets (h‐BN), which simultaneously functions as both a gel electrolyte and a separator, enabling maximum proximity between the positive and negative yarn‐based electrodes, subsequently enhancing the total energy storage efficacy of the device. The length‐matched asymmetric YSCs demonstrate excellent device aerial (gravimetric) capacitance of ≈658 mF cm^−2^ (≈53 F g^−1^) with high cyclic stability (≈76%, 10 000 cycles) and a large voltage window of 1.3 V.

We also present a simple and innovative approach for integrating asymmetric YSCs into textiles using common rivets readily used in existing garments, such as denim jackets, to serve as click‐on/off buttons, enabling toggled charge/discharge of YSCs. A proof‐of‐concept integration of asymmetric YSCs via snap rivets for rapid power delivery is demonstrated, allowing multiple YSCs to be interconnected in series and parallel to enhance voltage and capacitance as needed. The integrated energy textile showed the real‐world applicability of asymmetric YSCs by powering a digital clock and a light emitting diode (LED). The use of riveted interconnection enables the disassembly of the textile layer integrated with YSCs, allowing for safe handling of the E‐textiles during washing. The design also includes a concealing textile cover layer for both aesthetics and protection of the functional components. The advancements presented in this work mark a significant leap toward the practical application of YSCs as energy storage solutions for future E‐textiles and other flexible, portable, and wearable electronic technologies.

## 2 Results and Discussion

### 2.1 Chemical, Structural, Optical, and Morphological Characterization

**Figure 2:**
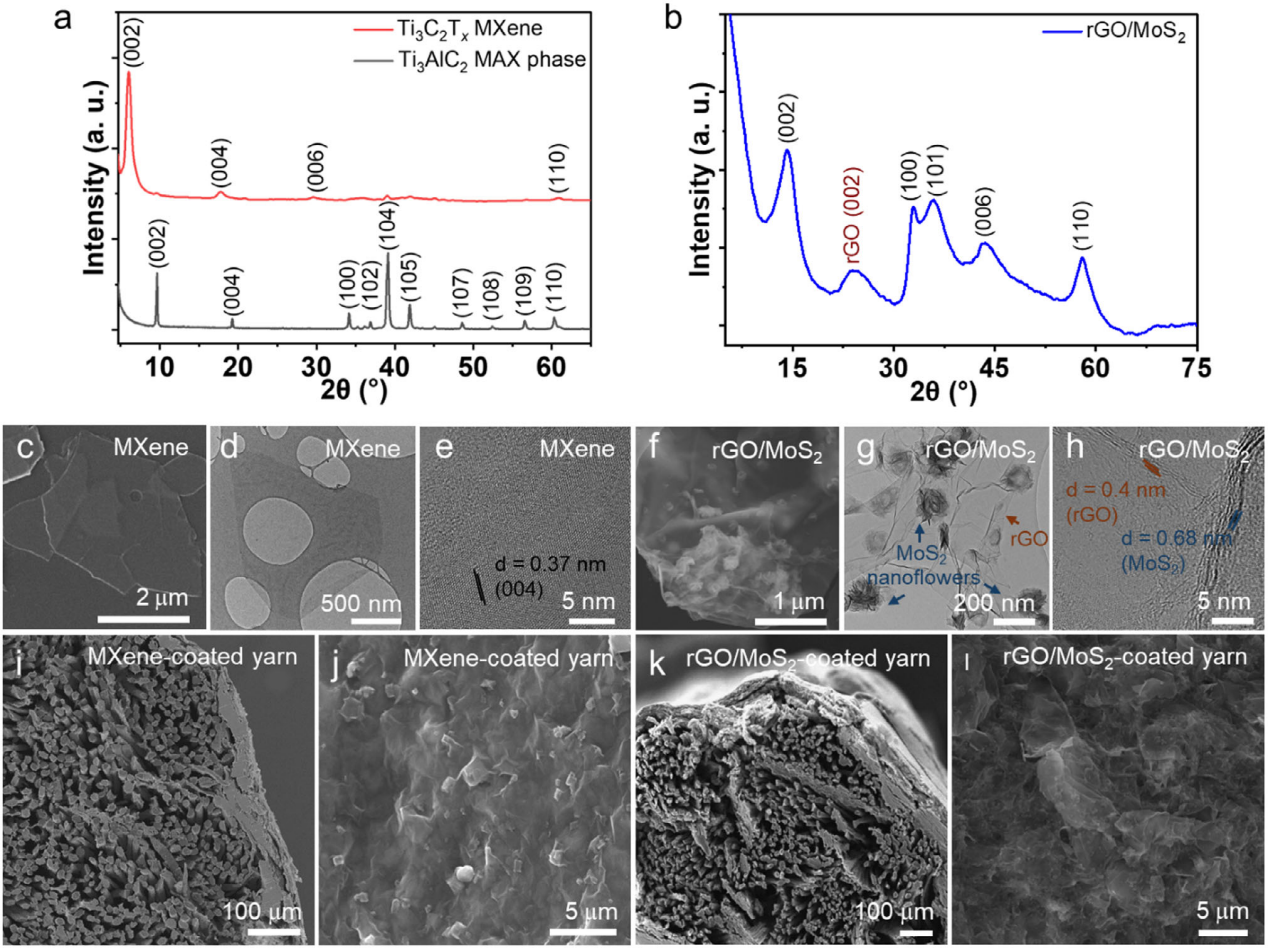
a) XRD of Ti_3_C_2_T_*x*_ MXene and Ti_3_AlC_2_ MAX phase, b) XRD of rGO/MoS_2_, c) SEM image, and d,e) TEM images of Ti_3_C_2_T_*x*_ MXene. f) SEM image and g,h) TEM images of rGO/MoS_2_. SEM images of i) cross‐section and j) surface morphology of Ti_3_C_2_T_*x*_ MXene‐coated yarn. SEM images of k) cross‐section and l) surface morphology of rGO/MoS_2_‐coated yarn.

We synthesized Ti_3_C_2_T_*x*_ MXene as the active material for the development of negative yarn electrode for asymmetric YSCs by selectively etching the Al layer in Ti_3_AlC_2_ MAX phase using the minimally intensive layer delamination (MILD) method.^[^ ^30^ ^]^ Structural characterizations using X‐ray diffraction (XRD) revealed successful etching of the Al layer in the Ti_3_AlC_2_ MAX phase, successfully forming highly delaminated Ti_3_C_2_T_*x*_ MXene, observed by the disappearance of the (104) diffraction peak of the MAX phase at the 2*θ* of ≈39° and downshifting of the (002) diffraction peak from the 2*θ* of ≈9.7° for the MAX phase to ≈5.9° for MXene (**Figure** **2**a).^[^ ^31^ ^]^ Subsequently, the interlayer spacing of ≈9.4 Å in the Ti_3_AlC_2_ MAX phase increased to ≈14.97 Å in Ti_3_C_2_T_*x*_ MXene, indicating the delamination of single‐layer MXene flakes. Additionally, the peak corresponding to the higher‐order (004) plane reflection at the 2*θ* of ≈17.7° in Ti_3_C_2_T_*x*_ MXene indicated the periodic flake arrangement within the layered material and further confirmed the delamination of Ti_3_C_2_T_*x*_ MXene. We also synthesized rGO/MoS_2_ nanohybrids as active materials combining both EDLC (from rGO) and pseudocapacitance (from MoS_2_) for the development of positive yarn electrodes for asymmetric YSCs. The synthesis used a hydrothermal approach to grow MoS_2_ onto GO flakes while in situ reducing the GO. The abundant oxygen‐rich functional groups (—OH, —COOH, and —O—) of GO enabled its use as a favorable substrate for the hydrothermal growth of MoS_2_ by facilitating MoS_2_ nucleation by providing anchoring sites for Mo and S precursors. The XRD of hydrothermally produced rGO/MoS_2_ (Figure 2b) showed distinct diffraction peaks at 14.2°, 32.8°, 35.8°, 43.4°, and 58.1°, corresponding to the (002), (100), (103), (105), and (110) planes of MoS_2_ 2 H phase (JCPDS No. 00‐037‐1492), respectively, confirming the MoS_2_ was successfully integrated with the rGO sheets to form an rGO/MoS_2_ nanohybrid.^[^ ^32^ ^]^ Additionally, the broad peak at ≈24.2° corresponded to the (002) plane of rGO, indicating its in situ formation during the hydrothermal synthesis of the rGO/MoS_2_ nanohybrid at 185 °C. We also prepared h‐BN nanosheets using an alkali‐assisted hydrothermal approach for use as a separator. The XRD pattern of h‐BN nanosheets (Figure S1, Supporting Information) showed diffraction peaks at 26.4°, 41.5°, 43.6°, 54.9°, and 75.8°, corresponding to the (002), (100), (101), (004), and (110) planes, respectively, confirming the successful formation of h‐BN (JCPDS No. 34‐0421).^[^ ^33^ ^]^

We further characterized our negative and positive electrode materials using Raman spectroscopy. The Raman spectrum of Ti_3_C_2_T_*x*_ MXene showed two key spectral regions (Figure S2a, Supporting Information), that is, a low‐frequency region (100–800 cm^−1^) representing lattice vibrations or phonons and a mid‐frequency region (1 000–1 800 cm^−1^) associated with C—C stretching vibrations within the carbon structures present in Ti_3_C_2_T_*x*_ MXene. Notably, the peak at ≈371 cm^−1^ was assigned to the in‐plane vibrations of planar Ti—C in Ti_3_C_2_T_*x*_ MXene.^[^ ^34^ ^]^ Raman bands related to surface groups of MXene, such as Ti_3_C_2_F_2_ and Ti_3_C_2_(OH)_2_, were also present at ≈255 and ≈448 cm^−1^, respectively. The Raman spectrum of rGO/MoS_2_ (Figure S2b, Supporting Information) exhibited two characteristic peaks of hexagonal‐MoS_2_ in the range of 365–406 cm^−1^. These bands (≈403 and ≈370 cm^−1^) corresponded to the out‐of‐plane (^1^A_g_) vibrational modes of sulfur atoms and the in‐plane (^1^E_2g_) vibrations of sulfur atoms on molybdenum, respectively.^[^ ^35^ ^]^ Additionally, the distinct peaks in the carbon spectral region at ≈1 352 and ≈1 588 cm^−1^ were assigned to the D and G bands, respectively, confirming the presence of graphitic carbon from rGO. We also carried out X‐ray photoelectron spectroscopy (XPS) analyses of Ti_3_C_2_T_*x*_ MXene and rGO/MoS_2_ electrode materials (Figure S3, Supporting Information). The XPS of Ti_3_C_2_T_*x*_ MXene revealed characteristic peaks corresponding to Ti 2p, C 1s, O 1s, and F 1s, confirming the Ti_3_C_2_T_*x*_ MXene formation with surface terminations, such as —O, —OH, and —F. Notably, the Ti 2p spectrum exhibited doublets at ≈455 and ≈462 eV, corresponding to Ti 2p_3/2_ and Ti 2p_1/2_, respectively, typically associated with Ti—C and Ti—O/F bonds.^[^ ^36^ ^]^ The XPS of rGO/MoS_2_ showed the characteristic peaks for C 1s, O 1s, Mo 3 d, and S 2p, confirming the presence of rGO and MoS_2_. Notably, the Mo 3 d spectrum of MoS_2_ displayed well‐resolved doublet peaks at ≈228.6 eV (3d_5/2_) and ≈231.9 eV (3d_3/2_), indicating the presence of Mo (IV) oxidation state, while the C 1s spectrum showed carbon bonding states, reflecting the hybrid structure.^[^ ^35^ ^]^

We also measured a Zeta potential of −40.5 mV for Ti_3_C_2_T_*x*_ MXene and −35.3 mV for rGO/MoS_2_ aqueous dispersions at a pH of 6.9 revealing negatively charged and hydrophilic surfaces that originated from the surface/terminal functional groups, enabling their use as coatings on cotton yarns (Figure S4, Supporting Information). Ti_3_C_2_T_*x*_ MXene flake size investigation using dynamic light scattering (DLS), revealed medium flake sizes ranging between ≈500 nm and ≈1 μm with an average hydrodynamic diameter of ≈754 nm (Figure S5, Supporting Information). We tailored our synthesis and processing to favor small Ti_3_C_2_T_*x*_ MXene flake sizes as they have previously been shown to lead to higher performance when used as electrodes in supercapacitors.^[^ ^37^ ^]^ Further characterizations using ultraviolet–visible (UV–Vis) spectroscopy revealed the signatures of Ti_3_C_2_T_*x*_ MXene (Figure S6a, Supporting Information), identified by two distinct UV peaks at ≈260 and ≈325 nm and broad absorption in the near‐infrared range at ≈755 nm.^[^ ^37^ ^]^ Notably, the UV peak at ≈260 nm was more prominent indicating the dominance of smaller flakes with more surface terminations, corroborating the DLS results. The UV–Vis spectrum of rGO/MoS_2_ (Figure S6b, Supporting Information) showed a peak at ≈216 nm, corresponding to the π–π* transitions of C=C bonds and a shoulder peak at ≈300 nm, resulting from the residual oxygen functionalities of rGO.^[^ ^35^ ^]^ The signatures of MoS_2_ were also present in the UV–Vis spectrum of rGO/MoS_2_, which included a peak at ≈461 nm, resulting from interband transitions between the occupied orbital (d_z_^2^) and the unoccupied orbitals (dxy, d*x* ^2^–*y* ^2^, and dxz,yz) of MoS_2_,^[^ ^38^ ^]^ and broad absorption in the 600–700 nm range, corresponding to the excitonic interband transitions in MoS_2_.^[^ ^39^ ^]^

The morphological observation of Ti_3_C_2_T_*x*_ MXene using scanning electron microscopy (SEM, Figure 2c) revealed highly exfoliated flakes shown by their high transparency together with minimal microscopic defects, indicating the successful etching of the Ti_3_AlC_2_ MAX phase into highly delaminated Ti_3_C_2_T_*x*_ MXene. Additionally, transmission electron microscopy (TEM) analysis further confirms the formation of delaminated Ti_3_C_2_T_*x*_ MXene flakes (Figure 2d). The size of MXene flakes, as determined from SEM and TEM measurements, ranged from ≈800 nm to ≈4 μm, corroborating the DLS result. The high‐resolution TEM (HRTEM) image (Figure 2e) of a typical Ti_3_C_2_T_*x*_ MXene flake showed fringes that were ≈0.37 nm apart and matched with the d‐spacing of the (004) plane, supporting the XRD results.^[^ ^40^ ^]^ The SEM images of rGO/MoS_2_ (Figure 2f) revealed the presence of a few‐layered MoS_2_ nanoflowers, intricately integrated onto the rGO flakes which acted as supporting envelopes (also see Figure S7a, Supporting Information). The TEM image of rGO/MoS_2_ confirmed the presence of MoS_2_ nanoflowers with hollow morphology (Figure 2g). Further morphological investigations using HRTEM also confirmed the distinct hollow structure of the MoS_2_ nanoflowers, showing MoS_2_ flakes comprised of 3–15 layers (Figure S7b,c, Supporting Information). The HRTEM image of a typical rGO/MoS_2_ nanohybrid exhibited an interlayer spacing of ≈0.68 nm, corresponding to the (002) plane of MoS_2_ 2 H phase (Figure 2h). The interlayer spacing of ≈0.4 nm was assigned to the (002) plan of rGO, indicating the partially restored graphitic structure with some residual oxygen functionalities. The strain induced by the curled morphology facilitated the formation of the 2 H phase.^[^ ^41^ ^]^ Notably, the MoS_2_ nanoflakes grew uniformly on the surface of the large GO flakes. The rGO nanosheet framework in the rGO/MoS_2_ nanohybrid could play a crucial role in efficiently delivering electrons to the active MoS_2_ sites. This allows for minimizing the undesirable potential drop (i.e., Ohmic loss) caused by the inherently poor electrical conductivity of MoS_2_, enhancing the electrode's performance. The MoS_2_ nanoflowers in this work were also considerably smaller in size (with a diameter in the range of 80–200 nm), compared to the previously reported MoS_2_ nanoflowers.^[^ ^42^ ^]^ These small size of nanoflowers offer abundant edge sites on the MoS_2_ nanosheets, which could be beneficial for supercapacitor application by acting as active sites for surface redox pseudocapacitance.

We also prepared free‐standing Ti_3_C_2_T_*x*_ MXene films using vacuum‐assisted filtration. The Ti_3_C_2_T_*x*_ MXene films showed an excellent electrical conductivity of ≈8 218 S cm^−1^, comparable to the previously reported values.^[^ ^15^, ^43^ ^]^ While, rGO/MoS_2_ films exhibited a lower electrical conductivity of ≈133 S cm^−1^, this value was higher than the electrical conductivities reported in the literature for rGO/MoS_2_, which range from ≈0.4 to 118 S cm^−1^.^[^ ^44^ ^]^ The excellent electrical conductivity of Ti_3_C_2_T_*x*_ MXene and rGO/MoS_2_ indicated their suitability for use as a negative and positive electrode, respectively, for constructing asymmetric YSCs.

### 2.2 Negative and Positive Electrode Yarns for YSCs

We used a facile, cost‐effective, and scalable dip‐coating process to integrate the negative (Ti_3_C_2_T_*x*_ MXene) and positive (rGO/MoS_2_) electrode materials onto multistrand cotton yarns. The as‐synthesized Ti_3_C_2_T_*x*_ MXene aqueous dispersion was suitable for coating cotton yarns upon adjusting the concentration (≈25–30 mg mL^−1^) to achieve sufficient viscosity (≈60 Pa s). Given the hydrophobic nature of the rGO/MoS_2_ nanohybrid and the lack of compatibility with cotton yarns, we prepared a viscous dispersion of rGO/MoS_2_ in dimethyl sulfoxide (DMSO) using a solvent exchange approach, enabling an effective and uniform coating on the cotton yarn. By repeating the dip‐coating process ten times, we achieve a maximum mass loading of ≈45 wt.% for Ti_3_C_2_T_*x*_ MXene and 13 times to reach ≈55 wt.% for rGO/MoS_2_ in cotton yarns. The XRD spectra of the Ti_3_C_2_T_*x*_ MXene‐ and rGO/MoS_2_‐coated yarns clearly showed the characteristic peak at 2θ of ≈22.6° for cotton as well as the signature peaks of Ti_3_C_2_T_*x*_ MXene and rGO/MoS_2_ active electrode materials, respectively, confirming the successful coatings (Figure S8, Supporting Information). The SEM image of an uncoated cotton yarn (Figure S9a, Supporting Information) revealed the presence of many filaments as well as capillary openings through which Ti_3_C_2_T_*x*_ MXene or rGO/MoS_2_ dispersions infiltrated to access the inner filaments. Dipping the hydrophilic cotton yarns with negatively charged Ti_3_C_2_T_*x*_ MXene and rGO/MoS_2_ resulted in uniform coatings that established continuous and interconnected networks of overlapping flakes along the surface of the cotton yarn (Figure 2i,j, and S9a, Supporting Information). Notably, coatings were achieved both onto the exterior and interior surfaces (e.g., filaments) of the yarn. Further analysis using energy dispersive X‐ray spectroscopy (EDS) confirmed the presence of Ti_3_C_2_T_*x*_ MXene and rGO/MoS_2_ coating layers on the surface of cotton (C and O) filaments in the negative (Ti, C, O, and F) and positive electrode yarns (Mo and S), respectively (Figure S10, Supporting Information). A closer observation of the surface of rGO/MoS_2_‐coated yarn revealed a porous structure containing microscale openings on the surface (Figure 2k,l). This porosity is highly desirable for EDLC. Additionally, the morphological features of the original active electrode material were retained during the coating process. For instance, nanoflowers of MoS_2_ anchored onto the rGO flakes remained intact after coating (Figure 2l). This effective coating process was facilitated by the strong electrostatic interactions between the electrode active materials and the filaments in the cotton yarn. The average thickness of the Ti_3_C_2_T_*x*_ MXene coating on the yarn surface was ≈25–35 μm (Figure 2i), while the rGO/MoS_2_ coating was measured as ≈25–40 μm (Figure 2k).

We measured the mechanical properties of the Ti_3_C_2_T_*x*_ MXene‐ and rGO/MoS_2_‐coated cotton yarns (Figure S11 Supporting Information). Uniaxial tensile testing on the Ti_3_C_2_T_*x*_ MXene‐coated cotton yarn (45wt%) revealed a Young's modulus of ≈1.14 GPa, a tensile strength of ≈129.2 MPa, and an elongation at break of ≈10.08% which were ≈23%, ≈10%, and 14% higher than those of the pristine cotton yarns, respectively. These improved mechanical properties could be attributed to the interactions of Ti_3_C_2_T_*x*_ MXene flakes with cotton yarns giving rise to improved structural integrity. While the rGO/MoS_2_‐coated cotton yarn (50 wt% mass loading with ≈67.5 wt% MoS_2_) exhibited slightly reduced mechanical properties (Young's modulus of ≈453.3 MPa, tensile strength of ≈48.8 MPa, and elongation at break of ≈7.7%) relative to the pristine cotton yarns, they still possess suitable mechanical properties for wearable applications. This decrease in mechanical properties could be attributed to the porous surface morphology induced by post‐coating chemical treatments (i.e., further GO reduction using hydrazine hydrate), which likely weakened the cotton yarn.

The electrical conductivity of Ti_3_C_2_T_*x*_ MXene‐ and rGO/MoS_2_‐coated cotton yarns increased with the active material loading (Figure S12 Supporting Information). This increase in electrical conductivity originated from a reduction in the overall resistance of the yarn with the coating additional layers of active materials, contributing toward the formation of new conduction pathways. The Ti_3_C_2_T_*x*_ MXene‐coated yarns showed a maximum electrical conductivity of ≈175.8 S cm^−1^ at the Ti_3_C_2_T_*x*_ MXene loading of ≈75 wt%. This conductivity is comparable to the previously reported values for Ti_3_C_2_T_*x*_ MXene‐based yarns.[^8^, ^17^, ^45^] For the rGO/MoS_2_‐coated yarns, we achieved a maximum electrical conductivity of ≈70.3 S cm^−1^ at the rGO/MoS_2_ loading of ≈70 wt%. We noted a decrease in the flexibility of the coated yarns with increasing the active material loading. The Ti_3_C_2_T_*x*_ MXene‐coated yarn at ≈45 wt.% (electrical conductivity of ≈44.9 S cm^−1^) and the rGO/MoS_2_‐coated yarn at ≈50 wt.% (electrical conductivity of ≈10.4 S cm^−1^) represented the balanced electrical conductivity and flexibility for use as the electrodes for YSCs. The lower electrical conductivities achieved for the coated yarn electrodes compared to the conductivities of the pristine free‐standing films could be attributed to the relatively low active material loadings in the yarns (compared to the pristine samples), the use of small‐size flakes to favor electrochemical properties, the highly porous nature of the cotton yarn, and the interface resistance from the insulating cotton fiber substrates.

**Figure 3:**
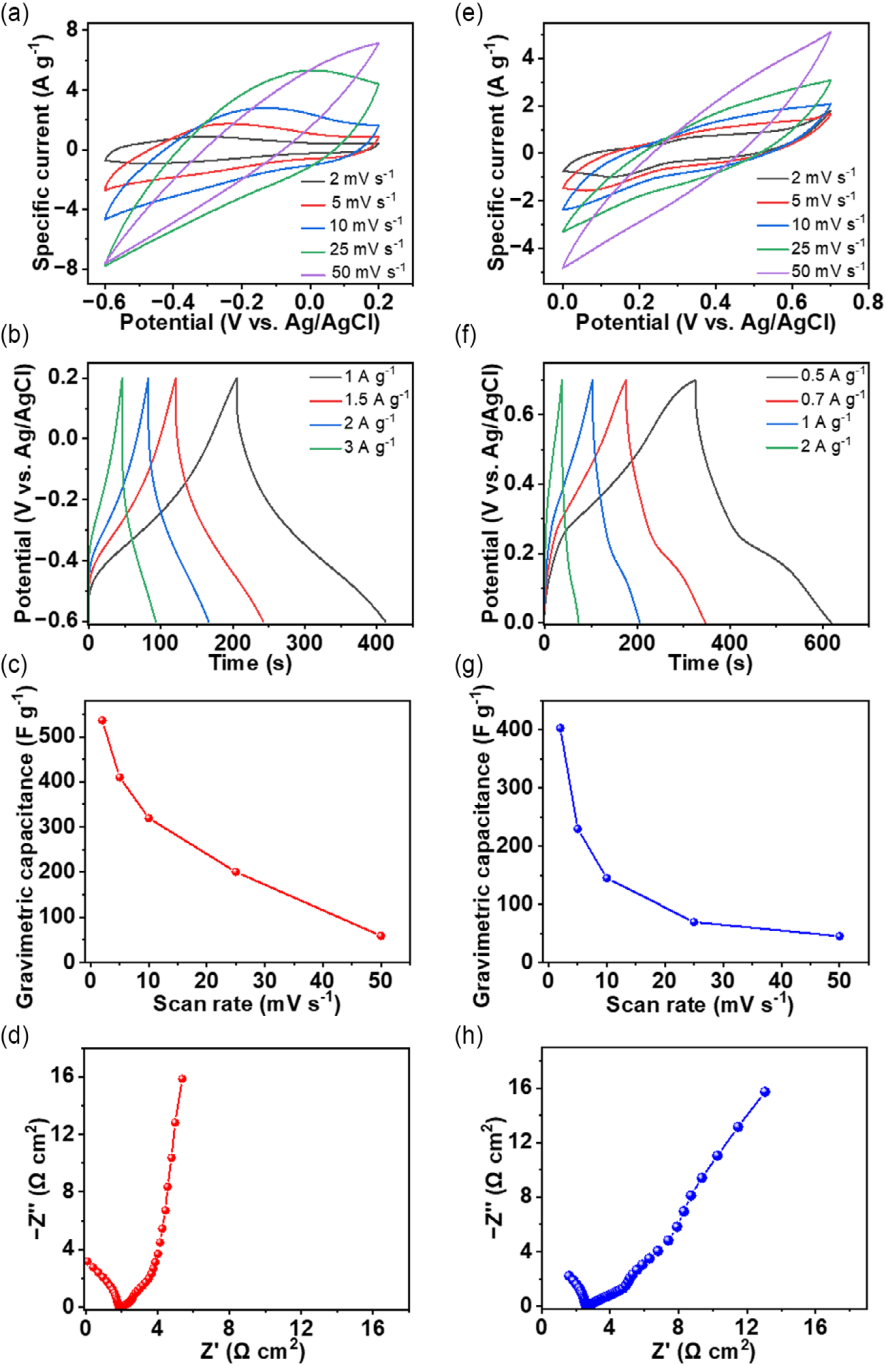
Electrochemical testing results for the Ti_3_C_2_T_*x*_ MXene‐coated cotton yarn (≈45 wt% loading): a) CV curves at different scan rates, b) GCD curves at different current densities, c) rate capability performance, and d) Nyquist plot obtained from EIS. Electrochemical testing results for the rGO/MoS_2_‐coated cotton yarn (≈55 wt% loading with ≈67.5 wt% MoS_2_): e) CV curves at different scan rates, f) GCD curves at different current densities, g) rate capability performance, and h) Nyquist plot obtained from EIS.

We investigated the electrochemical properties of the Ti_3_C_2_T_*x*_ MXene‐ and rGO/MoS_2_‐coated yarns in a three‐electrode cell using 1M H_2_SO_4_ as an electrolyte (Figure S13, Supporting Information) to evaluate their suitability for use as the negative and positive electrodes, respectively, for the development of YSCs. The cyclic voltammetry (CV) behaviors of Ti_3_C_2_T_*x*_ MXene‐coated yarns at various scan rates (2–50 mV s^−1^) showed rectangular‐like shapes and broad anodic and cathodic peaks between −0.6 and 0.2 V (vs. Ag/AgCl), which were more noticeable at low scan rates (**Figure** **3**a). The shape of the CV curves and the operating potential window observed in our work corroborate with the literature results for Ti_3_C_2_T_*x*_ MXene.^[^ ^46^ ^]^ The broad anodic and cathodic peaks in the CV behaviors correspond to H^+^ intercalation/deintercalation and surface redox reactions of Ti_3_C_2_T_*x*_ MXene.^[^ ^47^ ^]^ We investigated the effect of Ti_3_C_2_T_*x*_ MXene loading by estimating the gravimetric (based on the mass of the active electrode materials), areal, and length specific capacitances from the CV curves at a scan rate of 2 mV s^−1^. We noted that the gravimetric capacitance of the Ti_3_C_2_T_*x*_ MXene‐coated yarn at 2 mV s^−1^ increased initially with the Ti_3_C_2_T_*x*_ MXene loading and peaked at ≈536 F g^−1^ (areal and length capacitances of ≈7 360 mF cm^−2^ and ≈1 942 mF cm^−1^) for ≈45 wt.% Ti_3_C_2_T_*x*_ MXene loading and then decreased (Figure S14a, Supporting Information). This unexpected decrease in gravimetric capacitance was caused because of a lower interaction of the Ti_3_C_2_T_*x*_ MXene flakes and the surface of the cotton filaments with the increase in the thickness of the coating layer and a subsequent flake‐off, which reduced the available active sites for surface redox reactions to occur. Consequently, the Ti_3_C_2_T_*x*_ MXene‐coated yarn with ≈45 wt.% MXene loading was the most suitable yarn composition for the negative electrode. The specific capacitance (≈7,360 mF cm^−2^ and ≈1,942 mF cm^−1^ at 2 mV s^−1^) of the Ti_3_C_2_T_*x*_ MXene‐coated yarn in this work is among the highest reported for yarn‐based supercapacitor electrodes, outperforming previously reported values, such as biscrolled Ti_3_C_2_T_*x*_ MXene/CNT yarn (≈3,188 mF cm^−2^ and ≈118 mF cm^−1^),^[^ ^21^ ^]^ Ti_3_C_2_T_*x*_ MXene onto Z‐directional fiber bundles (≈1,749 mF cm^−2^),^[^ ^48^ ^]^ Ti_3_C_2_T_*x*_ MXene‐coated wool yarn (≈1,854 mF cm^−2^),^[^ ^49^ ^]^ Ti_3_C_2_T_*x*_ MXene silver‐plated nylon yarn (≈253 mF cm^−1^),^[^ ^50^ ^]^ and Ti_3_C_2_T_*x*_ MXene‐coated cellulose yarn (≈760 mF cm^−1^).^[^ ^17^ ^]^

The galvanostatic charge–discharge (GCD) curves at various current densities (1–3 A g^−1^) showed symmetrical and triangular shapes, even at the high current density of 3 A g^−1^ (Figure 3b). The discharge time increased with decreasing the current density, whereby, for the Ti_3_C_2_T_*x*_ MXene‐coated yarn at ≈45 wt.%, a maximum discharge time of ≈210 s was achieved at 1 A g^−1^. Using GCD, we measured the gravimetric, areal, and length capacitances of ≈262 F g^−1^, ≈3,595 mF cm^−2^, and ≈949 mF cm^−1^, respectively, at the current density of 1 A g^−1^ (equivalent to 13.7 mA cm^−2^ and 3.6 mA cm^−1^). The lower specific capacitance compared to those measured based on CV was attributed to the high current densities used during the GCD measurements. The rate capability investigation using CV (Figure 3c) showed an overall decrease in the gravimetric capacitance of the Ti_3_C_2_T_*x*_ MXene‐coated yarn with the scan rate, while the sample still showed a high gravimetric capacitance of ≈200.8 F g^−1^ as the scan rate of 25 mV s^−1^. The observed decrease in specific capacitance with scan rate is due to the diffusion limitations of ionic transport, a trend also observed in previous studies on thick planar Ti_3_C_2_T_*x*_ MXene electrodes.^[^ ^17^ ^,^ ^51^ ^]^

Further analysis of the capacitive contribution by fitting the anodic peak current of the CV curves versus scan rate (logarithmic scales) for the Ti_3_C_2_T_*x*_ MXene‐coated cotton yarn electrode revealed a slope (*b*‐value) of 0.65, indicating both diffusion‐controlled and capacitive contributions (Figure S15a, Supporting Information). We also used electrochemical impedance spectroscopy (EIS) to evaluate the suitability of the 45 wt.% Ti_3_C_2_T_*x*_ MXene‐coated yarn. The Nyquist plot obtained from EIS (Figure 3d) did not show the typical semicircle region at the high‐frequency regions, indicating a small charge transfer resistance. We measured a very low equivalent series resistance (ESR) of ≈1.76 Ω cm^2^, which indicated small intrinsic resistances for the electrode and electrolyte as well as efficient charge transport within the Ti_3_C_2_T_*x*_ MXene network deposited on the cotton filaments. The charge transfer resistance (*R*_ct_) was estimated as ≈4.09 Ω cm^2^, suggesting fast electrochemical kinetics at the electrode–electrolyte interface and the presence of active redox or pseudocapacitive processes. In the low‐frequency range, where Warburg‐type impedance dominated, an almost vertical line (with a slope of ≈90°) was observed which demonstrated an ideal capacitive behavior of the electrode. A similar shape of the Nyquist plot was observed for the Ti_3_C_2_T_*x*_ MXene/cellulose nanofiber electrode.^[^ ^52^ ^]^

To achieve a high‐performance positive yarn electrode that closely matched the specific capacitance of the Ti_3_C_2_T_*x*_ MXene‐coated negative electrode yarn, we synthesized rGO/MoS_2_ nanohybrids with various compositions (MoS_2_ content ranging from ≈34.9 to ≈71.7 wt.% MoS_2_) and varied the loading (≈40 to ≈70 wt.%) of active material (rGO/MoS_2_) on the cotton yarns. The electrochemical investigation of the rGO/MoS_2_‐coated cotton yarns using CV (Figure 3e) at various scan rates (2–50 mV s^−1^) showed a quasi‐rectangular and symmetrical shape in the potential range of 0–0.7 V (vs. Ag/AgCl). Contributions from both EDLC (from rGO) and surface redox pseudocapacitance (from MoS_2_) were present in the CV curves noted by the nearly rectangular shape of the CV curves and the presence of broad anodic and cathodic peaks at low scan rates (Figure 3e). These observations corroborate with the previously reported CV curves for rGO/MoS_2_.^[^ ^26^, ^27^ ^]^ Using the CV curves at the scan rate of 2 mV s^−1^, we estimated the gravimetric, areal, and length specific capacitances of the rGO/MoS_2_‐coated cotton yarns prepared with different MoS_2_ contents (≈34.9 to ≈71.7 wt.%), while keeping the rGO/MoS_2_ loading in the cotton yarn constant (at ≈50 wt.%). The gravimetric capacitance of the yarns increased with the increase in MoS_2_ content and peaked at ≈402 F g^−1^ (areal and length capacitances of ≈4 756 mF cm^−2^ and ≈1 278 mF cm^−1^) for ≈67.5 wt.% (Figure S14a,b, Supporting Information). Notably, the rGO/MoS_2_‐coated yarn with ≈67.5 wt.% MoS_2_ showed a higher gravimetric capacitance than the yarn with ≈50 wt.% MoS_2_ at the same mass loading. Consequently, the cotton yarn coated with ≈50 wt.% loading of rGO/MoS_2_ containing ≈67.5 wt.% MoS_2_ was most suited as the positive electrode for YSCs. Notably, the gravimetric capacitance of this rGO/MoS_2_‐coated yarn was four times higher than that of the rGO‐coated cotton yarn (≈95 F g^−1^). The enhanced specific capacitance of the rGO/MoS_2_ material with the MoS_2_ content is attributed to the additional pseudocapacitance contributions provided by MoS_2_. The abundant dangling edge sites and defects in the MoS_2_ nanosheets provide many active sites for surface redox reactions to take place, giving rise to the specific capacitance of the nanohybrid electrode material.^[^ ^53^ ^]^ Additionally, the edge‐terminated small MoS_2_ flakes provide an enhanced exposure for H^+^ intercalation and deintercalation through spatially confined ion transport channels, compared to rGO.^[^ ^54^ ^]^ The decrease observed with increasing mass loading of the rGO/MoS_2_ in the coated yarn beyond ≈50 wt.% for the active material with ≈67.5 wt.% MoS_2_ is due to the reduced accessibility of the active MoS_2_ sites at high loadings. The specific capacitance of the rGO/MoS_2_‐coated yarn in this work is among the highest reported values to date for a similar system, such as MoS_2_/rGO (128–265 F g^−1^ and ≈8 mF cm^−2^),^[^ ^55^ ^]^ MoS_2_/graphene (248 F g^−1^),^[^ ^56^ ^]^ MoS_2_/amorphous carbon (167.3 F g^−1^),^[^ ^57^ ^]^ NiO/MoS_2_/rGO (≈7.38 mF cm^−2^),^[^ ^58^ ^]^ and MoS_2_@CNT/rGO (≈129 mF cm^−2^).^[^ ^59^ ^]^

The GCD curves of the rGO/MoS_2_‐coated cotton yarn positive electrode (≈50 wt.% mass loading with ≈67.5 wt.% MoS_2_) measured at various current densities (0.5–2 A g^−1^) revealed a slight deviation from the perfect triangular shape typically observed for the EDLC materials (Figure 3f). This result further highlighted the presence of pseudocapacitance, which came from MoS_2_. A maximum discharge time of ≈295 s was achieved at 0.5 A g^−1^. Based on the GCD results, we calculated gravimetric, areal, and length capacitances of ≈210 F g^−1^, ≈2,484 mF cm^−2^, and ≈668 mF cm^−1^ at the current density of 0.5 A g^−1^ (equivalent to 5.9 mA cm^−2^ and 1.6 mA cm^−1^) for the rGO/MoS_2_‐coated cotton yarn. The specific capacitance determined from GCD measurements was lower than that from CV measurements, which is attributed to the high current densities employed in the GCD. The rate capability investigation revealed a gravimetric capacitance of ≈69.4 F g^−1^ for the rGO/MoS_2_‐coated cotton yarn at the scan rate of 25 mV s^−1^ (Figure 3g). While this represented a notable decrease in specific capacitance, the rGO/MoS_2_‐coated cotton yarn still showed comparable specific capacitance to the rGO‐coated yarns at this higher scan rate. The decrease in specific capacitance with scan rate is a common phenomenon in both EDLC and pseudocapacitive materials though pseudocapacitors typically exhibit a more noticeable drop due to the slower kinetics of the Faradaic processes.^[^ ^60^ ^]^ This is mainly due to the limited ion diffusion, increased resistance, and reduced access to the active sites.^[^ ^60^ ^]^

When analyzed the CV for the capacitive contribution by fitting the anodic peak current versus scan rate (logarithmic scales) for the rGO/MoS_2_‐coated cotton yarn electrode, a slope (*b*‐value) of 0.5 was obtained, indicating a predominantly diffusion‐controlled charge storage (Figure S15b, Supporting Information). The Nyquist plot for the rGO/MoS_2_‐coated yarn obtained from EIS showed an incomplete semicircle with a very low ESR of ≈2.58 Ω cm^2^ in the high‐frequency region and a nearly vertical line in the low‐frequency region, confirming the suitability of the rGO/MoS_2_‐coated yarn for use as an electrode for YSCs (Figure 3h). *R*_ct_ was estimated as ≈11.45 Ω cm^2^ indicating a relatively slow electrochemical kinetics, due to the redox‐active sites of MoS_2_.

### 2.3 Symmetric YSCs

To further evaluate the performance of the Ti_3_C_2_T_*x*_ MXene‐ and rGO/MoS_2_‐coated yarn electrodes in supercapacitor devices, we first developed symmetric YSCs by assembling two similar electrodes in parallel separated by a layer of a gel electrolyte (Figure S16, Supporting Information). We developed PVA‐H_2_SO_4_/h‐BN with ≈7.5 wt% loading of h‐BN as a new gel electrolyte for YSCs. When compared to the commonly used PVA‐H_2_SO_4_ gel electrolyte, our PVA‐H_2_SO_4_/h‐BN showed a higher specific capacitance and rate performance in Ti_3_C_2_T_*x*_ MXene‐coated yarn‐based symmetric YSC (Figure S17, Supporting Information). This performance enhancement could be attributed to the improved ionic mobility, higher stability, and greater electrolyte–electrode interactions as the result of the higher proximity of the electrodes. The symmetric YSC fabricated using Ti_3_C_2_T_*x*_ MXene‐coated yarn electrodes showed a working voltage of 0.8 V and excellent device gravimetric and aerial capacitances of ≈55.5 F g^−1^ and ≈247.6 mF cm^−2^ (at 2 mV s^−1^), achieving a maximum energy density of ≈7.6 Wh kg^−1^ and a power density of ≈200 W kg^−1^ (Figure S18a–c, Supporting Information). Similarly, the symmetric YSC made using the rGO/MoS_2_‐coated yarn showed a working voltage of 0.7 V and high device gravimetric and aerial capacitances of ≈23 F g^−1^ and ≈139.6 mF cm^−2^ (at 2 mV s^−1^), translating into a maximum energy density of ≈1.4 Wh kg^−1^ and a power density of ≈140 W kg^−1^ (Figure S18d–f, Supporting Information). Overall, the energy storage performance of the symmetric YSC made using Ti_3_C_2_T_*x*_ MXene‐coated yarn electrodes was higher than the YSC made of the rGO/MoS_2_‐coated yarns, highlighting the need for a further step of performance matching for the development of asymmetric YSCs.

### 2.4 Asymmetric YSCs

**Figure 4:**
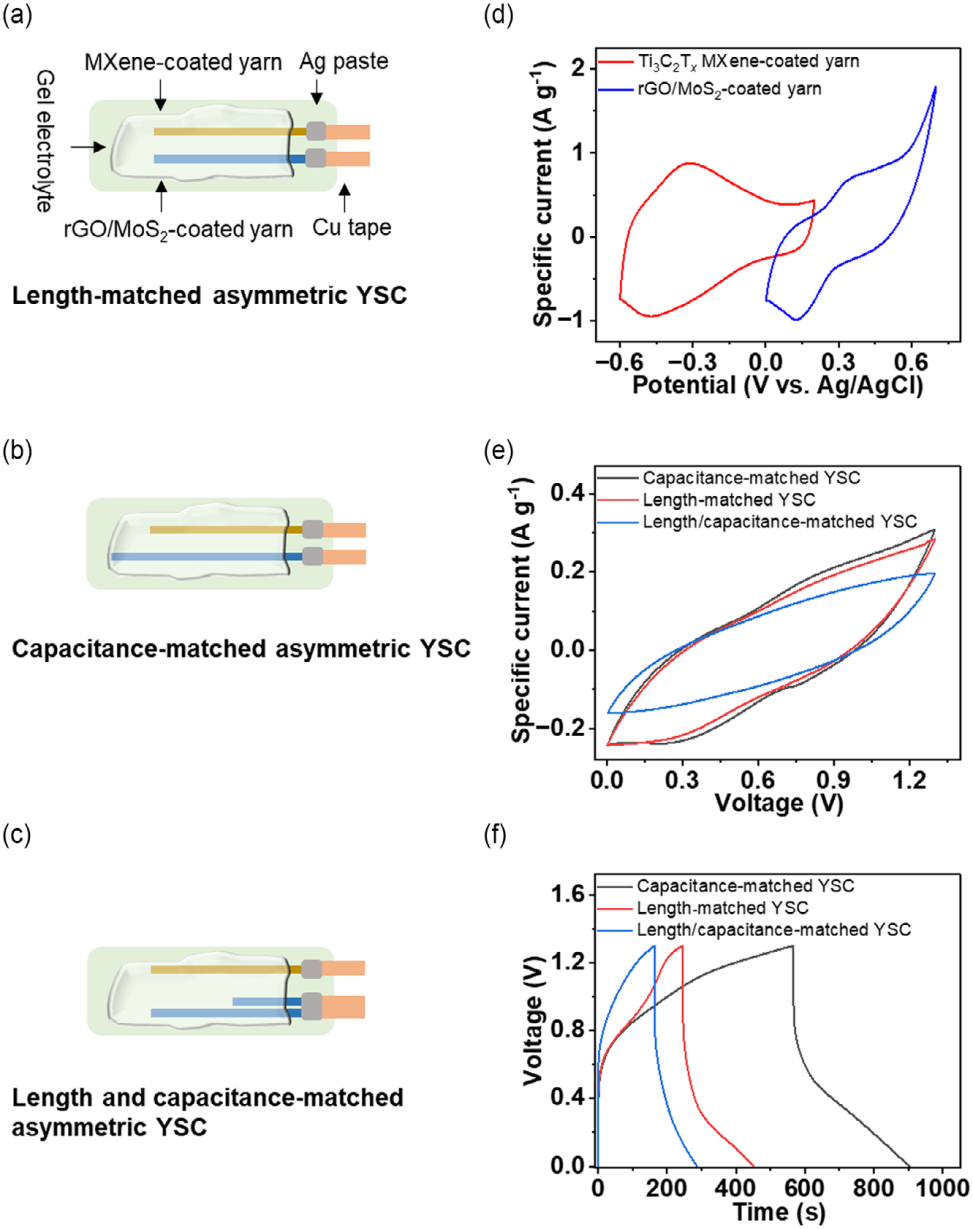
Schematic illustration of a) the length‐matched asymmetric YSC, b) the capacitance‐matched asymmetric YSC, and c) the length‐ and capacitance‐matched asymmetric YSC. d) CV curves of the Ti_3_C_2_T_*x*_ MXene‐coated negative electrode yarn and the rGO/MoS_2_‐coated positive electrode yarn measured at the scan rate of 2 Mv s^−1^, showing their operating potential ranges. e) CV and f) GCD curves of the length‐matched asymmetric YSC, capacitance‐matched asymmetric YSC, and length‐ and capacitance‐matched asymmetric YSC at the scan rate of 2 mV s^−1^ and the current density of 0.2 A g^−1^, respectively.

The working voltages achieved for the symmetric YSCs (0.7–0.8 V) are insufficient for powering electronic devices. Asymmetric YSCs are desirable as they provide a route to maximizing the voltage and energy density of supercapacitors. A critical aspect in achieving high performance and maximizing energy output in asymmetric YSCs is the maintenance of charge balance between the positive and negative electrodes (*Q*_+_ = *Q*_−_). As the specific capacitance and operating potential windows of the positive and negative electrodes can be different, charge balance can be achieved by adjusting the mass ratio between the positive and negative electrodes. Given the exceptionally high specific capacitance of the Ti_3_C_2_T_*x*_ MXene‐coated yarn (negative electrode), we carefully designed the rGO/MoS_2_‐coated cotton yarn (positive electrode) to achieve a specific capacitance (≈402 F g^−1^) that is reasonably close to that of the Ti_3_C_2_T_*x*_ MXene‐coated yarn (≈536 F g^−1^) allowing for the development of asymmetric YSCs. At a given mass loading, the mass (and volume) of the yarn electrode is proportional to its length, providing a facile approach to achieving perfect capacitance matching for asymmetric YSCs. This strategy is more feasible and controllable than active material mass adjustment, which requires careful control of active material deposition during electrode yarn production. Hence, to further account for and investigate the impact of the small shortfall in the specific capacitance of the rGO/MoS_2_‐coated cotton yarn, we employed various asymmetric YSC fabrication strategies to maximize the device performance. We carefully designed three types of asymmetric YSCs for comparison: 1) length‐matched YSC made with negative and positive electrodes of the same length (**Figure** **4**a), 2) capacitance‐matched YSC made with negative and positive electrodes of different lengths and matching capacitances via length adjustments (Figure 4b), and 3) length‐ and capacitance‐matched YSC made using an additional positive electrode yarn (Figure 4c). With an operating potential window range of −0.6–0.2 V (vs. Ag/AgCl) for the Ti_3_C_2_T_*x*_ MXene‐coated cotton yarn and 0–0.7 V (vs. Ag/AgCl) for the rGO/MoS_2_‐coated cotton yarn, they are ideally suited as the negative and positive electrodes, respectively, for the development of an asymmetric YSC that shows higher voltages than its symmetric counterparts (Figure 4d). All asymmetric YSCs used PVA‐H_2_SO_4_/h‐BN gel electrolyte.

The CV investigation showed that all three asymmetric YSCs operated with a working voltage of 1.3 V (Figure S19a,d,g, Supporting information). This is based on the overlapping potential ranges of Ti_3_C_2_T_*x*_ MXene‐coated yarn (−0.6–0.2 V (vs. Ag/AgCl) and rGO/MoS_2_‐coated yarns (0–0.7 V (vs. Ag/AgCl), spanning −0.6–0.7 V (vs. Ag/AgCl) (Figure 4d), which makes them well suited for the development of an asymmetric YSC that shows higher voltages than its symmetric counterparts. The CV curves of all three asymmetric YSCs showed a quasi‐rectangular shape with a pair of broad redox peaks, indicating the presence of the surface redox pseudocapacitance contribution of MoS_2_ (Figure 4e). Notably, the capacitance‐matched asymmetric YSC showed the highest area inside the CV curve. We measured the specific capacitance of ≈47 F g^−1^ (at 2 mV s^−1^) for the capacitance‐matched asymmetric YSC based on the CV results. The GCD results further supported the presence of pseudocapacitance from MoS_2_ by displaying a slight bulge within 0–1.3 V voltage range (Figure 4f). The comparison of different devices revealed that the capacitance‐matched asymmetric YSC had the longest discharge time of ≈341 s (at 0.2 a g^−1^), indicating its superior performance than the length‐matched and length‐ and capacitance‐matched YSCS. Based on the GCD results, we measured the gravimetric and areal capacitances of ≈53 F g^−1^ (at 0.2 a g^−1^) and ≈658 mF cm^−2^ (at 2.5 mA cm^−2^), respectively, for the capacitance‐matched asymmetric YSC. These were higher than the gravimetric and areal capacitances of ≈32 F g^−1^ (at 0.2 a g^−1^) and ≈421 mF cm^−2^ (at 2.6 mA cm^−2^) for the length‐matched asymmetric YSC and the gravimetric and areal capacitances of ≈19 F g^−1^ (at 0.2 a g^−1^) and ≈553 mF cm^−2^ (at 1.3 mA cm^−2^) for the length‐ and capacitance‐matched asymmetric YSC (Figure S19b,e,h, Supporting Information). This specific capacitance is amongst the highest reported value so far for asymmetric YSCs, such as rGO/CNT polyester yarn//Ni—Co bimetallic oxyhydroxide polyester yarn (≈133 mF cm^−2^),^[^ ^61^ ^]^ biscrolled Ti_3_C_2_T_*x*_ MXene/CNT//biscrolled RuO_2_/CNT yarn (≈554 mF cm^−2^),^[^ ^21^ ^]^ and Ti_3_C_2_T_*x*_ MXene coaxial zinc‐ion hybrid fiber (≈214 mF cm^−2^).^[^ ^62^ ^]^ Additionally, the capacitance‐matched asymmetric YSC showed excellent long‐term cyclic stability, maintaining ≈75.1% of its initial capacitance after 10 000 charge/discharge cycles with a consistent Coulombic efficiency of ≈100% throughout the cycles (Figure S19c, Supporting Information).This was higher than the capacitance retention of ≈66.5% (Coulombic efficiency of ≈100%) for the length‐matched asymmetric YSC and the capacitance retention of ≈64.34% (Coulombic efficiency of ≈92%) for the length‐ and capacitance‐matched asymmetric YSC tested under the same cyclic conditions (Figure S19f,i, Supporting Information).

We also measured the energy density and power density of different YSCs. Once again, the capacitance‐matched asymmetric YSC was proven to be superior with a maximum energy density of ≈12.3 Wh kg^−1^ (≈155 μW h cm^−2^) and a power density of ≈650 W kg^−1^ (≈8,147 μW cm^−2^) compared to the length‐matched asymmetric YSC (≈7.3 Wh kg^−1^ and ≈325 W kg^−1^) and the length‐ and capacitance‐matched asymmetric YSC (≈4.5 Wh kg^−1^ and ≈260 W kg^−1^). Notably, the energy density and power density of capacitance‐matched asymmetric YSCs were higher than the symmetric YSCs (made using two similar Ti_3_C_2_T_*x*_ MXene‐ or rGO/MoS_2_‐coated yarn electrodes). The energy density of capacitance‐matched asymmetric YSC developed in this work is among the highest reported in the literature including symmetric YSCs, such as Ti_3_C_2_T_*x*_ MXene@Ag coated nylon fiber (≈7.3 μWh cm^−2^),^[^ ^50^ ^]^ Ti_3_C_2_T_*x*_ MXene‐coated wool yarns (≈3.7 μWh cm^−2^),^[^ ^49^ ^]^ as well as asymmetric YSCs such as rGO/CNT polyester yarn//NiCo BOH polyester yarn (≈78.1 μWh cm^−2^),^[^ ^61^ ^]^ Ti_3_C_2_T_*x*_ MXene coaxial zinc‐ion hybrid fiber (≈42.8 μWh cm^−2^),^[^ ^62^ ^]^ biscrolled MXene/CNT//biscrolled RuO_2_/CNT yarn (≈167.9 μWh cm^−2^),^[^ ^21^ ^]^ and MnO_2_/CNT/graphene@Ni wire//Ni tube (≈3.1 μWh cm^−2^).^[^ ^63^ ^]^ This comparison was presented visually in a Ragone plot (Figure S20, Supporting Information), where the power density versus energy density trends were plotted for our capacitance‐matched asymmetric YSC in conjunction with the literature results. A comprehensive comparison of the specific capacitance, energy density, and power density for various YSCS was also provided in Table S1, Supporting Information. These results indicated that the conventional asymmetric YSC structure obtained by matching the lengths of the electrodes was ineffective and highlighted the need to match the capacitance of the electrodes to achieve maximized performance in asymmetric YSCS. We showed that this could be easily achieved by adjusting the length of the yarn electrodes.

### 2.5 Textile Integration of YSCs using Riveted Interconnection

**Figure 5:**
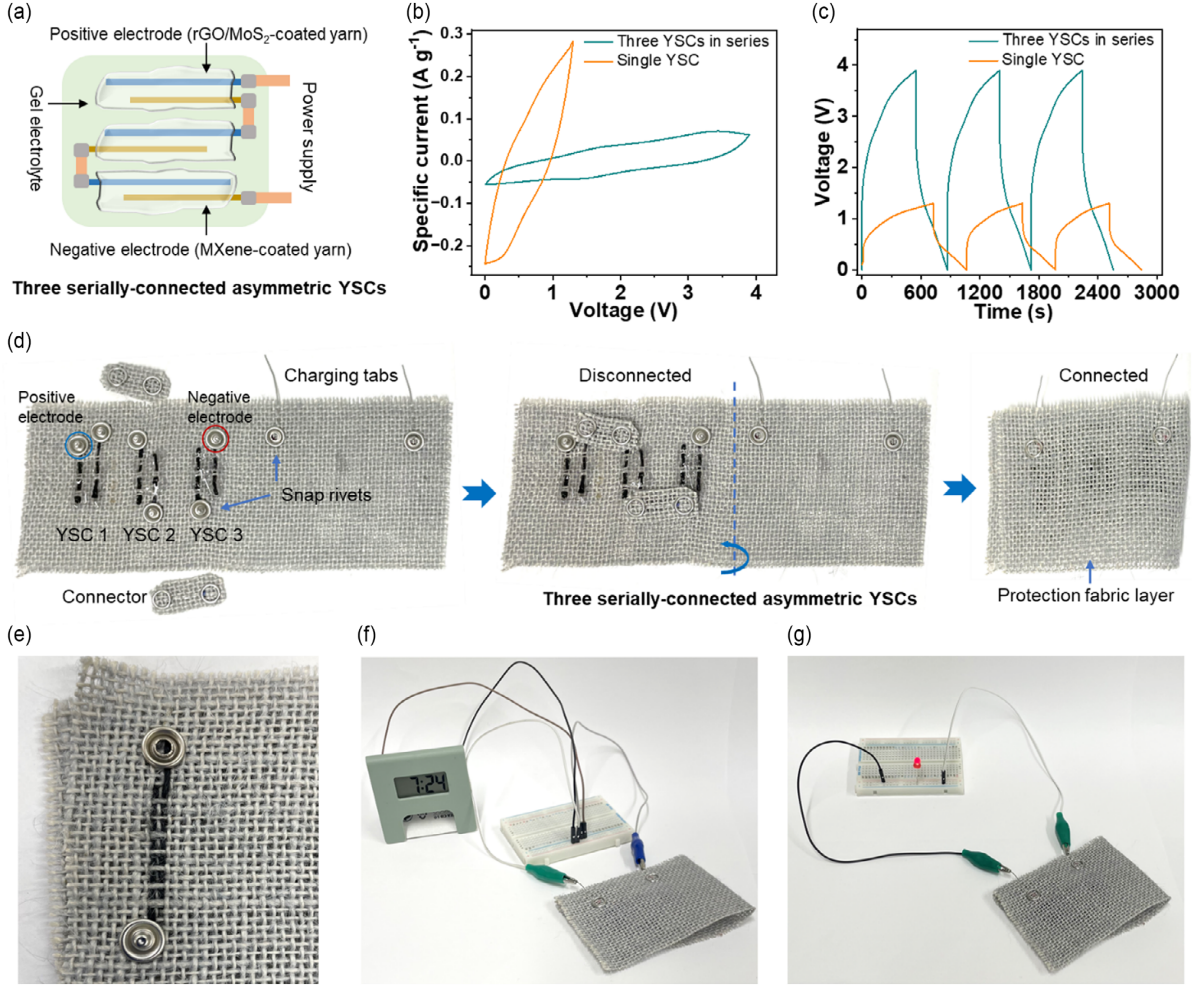
a) Schematic illustration of three serially‐connected asymmetric YSCs. Comparison of b) CV curves at 2 mV s^−1^ and c) GCD curves at 0.2 A g^−1^ for a single asymmetric YSC and three serially‐connected YSCs. Digital photographs showing d) integration of three serially‐connected asymmetric YSCs into a jute fabric using snap rivets, highlighting the connected and disconnected modes, e) close‐up view of a single asymmetric YSC integrated in the fabric using snap rivets allowing for the proximity of the electrodes, and the wearable energy storage fabric prototype powering f) a digital clock and g) a red LED.

Multiple asymmetric YSC devices can be electrically connected in series or parallel configurations to further enhance the voltage or energy output, respectively. By connecting three asymmetric YSCs in series (**Figure** **5**a), we achieved a working voltage of 3.9 V, which was three times the operating voltage of a single asymmetric YSC (1.3 V) as shown by CV (Figure 5b) and GCD (Figure 5c). This voltage is sufficient for powering a range of portable and wearable electronics, such as digital watches, medical sensors, electronic patches, and fitness trackers. We measured a gravimetric capacitance of ≈19.0 F g^−1^ at 2 mV s^−1^ along with an energy density of ≈35 Wh kg^−1^ for the in‐series asymmetric YSCs (only considering the masses of the active materials). Leveraging the large voltage window and the high energy density of the asymmetric YSC, we developed an energy‐storing fabric by incorporating three asymmetric YSCs connected in series to demonstrate their practicality to power off‐the‐shelf electronics. We employed an easy‐to‐use and novel design that took the advantage of metallic conductivity of snap rivets as both current collectors and charging tabs (Figure 5d). Snap rivets are commonly used in the textile industry, for instance, in denim jeans and jackets. We first sewed the Ti_3_C_2_T_*x*_ MXene‐coated yarns (negative electrodes) and the rGO/MoS_2_‐coated yarns (positive electrodes) into a piece of jute fabric at the desired positions. We then secured male snap rivets to one end of each yarn. Subsequently, we positioned female snap rivets strategically to allow for the establishment of tabs to supply or receive power. We also developed customized connectors, which we used to establish YSCs interconnections in series or parallel as necessary. The interconnected YSCs could then be put in the operation mode by simply connecting the male and female snap rivets. Once connected, the YSCs were safeguarded using a top fabric layer which also hid the YSCs providing improved aesthetics. The energy fabric containing YSCS can then be taken off by disconnecting the male snap rivets from the female ones. Using the riveted interconnection strategies enabled the energy fabric with YSCs to be easily detached from the fabric when not in use or during washing, ensuring consistent performance and extending the YSCs’ longevity. Additionally, the use of a two‐layer design prevented the YSCs from coming into direct contact with the wearer's body by providing an additional layer of fabric on the surface. Lastly, the use of riveted interconnection enabled the assembly of YSCs that benefitted from high proximity of yarn electrodes, employing only one weft yarn of the host fabric as a spacer (Figure 5e). We showed that the wearable energy storage fabric developed by stitching three interconnected asymmetric YSCs was able to power a digital clock (Figure 5f) for several hours and an LED (Figure 5g) for more than 30 s (recorded video, Supporting Information). Our riveted interconnection strategy is a simple approach to seamlessly integrate YSCs into existing textiles and requires minimal infrastructure adjustments as snap rivets are widely used in the textile industry, making this approach commercially viable and cost‐effective for mass production. The energy storage fabric developed in this work can be used to deliver on‐demand power for a range of wearable electronics, such as fitness bands, health monitoring sensors, and internet of things (IoT), taking advantage of the minimized device contact with the wearer's body, improved aesthetics, and washability.

We further evaluated the mechanical robustness of the asymmetric YSCs by measuring the CV behaviors under various bending angles of the energy storage fabric prototype (Figure S21 Supporting Information). The CV curves of the asymmetric YSCs showed minimal performance deviation during gradual bending up to 180° (specific capacitance decreased by only ≈8%), indicating that a stable electrochemical performance under various mechanical deformations. We also investigated the washability of the asymmetric YSC after two washing (15 min each with detergent) and drying (at 35 °C) cycles. The asymmetric YSC showed almost no change in its CV behavior and its specific capacitance remained unchanged after the two washing and drying cycles (Figure S22, Supporting Information), indicating washability and high environmental stability.

## 3 Conclusion

We fabricated a high‐performance asymmetric YSC by purposefully developing negative (Ti_3_C_2_T_*x*_ MXene‐coated cotton yarn) and positive (rGO**/**MoS_2_‐coated cotton yarn) electrodes with comparable specific capacitances and implementing a new device assembly based on matching the electrode capacitances as opposed to the traditional approach of matching length. The negative yarn electrode (Ti_3_C_2_T_*x*_ MXene‐coated cotton yarn) exhibited a maximum specific capacitance of ≈536 F g^−1^ (≈7 360 mF cm^−2^ and ≈1 942 mF cm^−1^) and the positive yarn electrode (rGO**/**MoS_2_‐coated cotton yarn) demonstrated a maximum capacitance of ≈402 F g^−1^ (≈4 756 mF cm^−2^ and ≈1 278 mF cm^−1^). We also developed a new PVA‐H_2_SO_4_/h‐BN gel electrolyte that allowed for the proximity of the yarn electrodes, enhancing the overall YSC's performance by providing long‐term stability and helping to prevent short circuits. The capacitance‐matched asymmetric YSC showed a maximum specific capacitance of ≈53 F g^−1^ (≈658 mF cm^−2^ and ≈177 mF cm^−1^) and excellent long‐term stability over 10 000 charge/discharge cycles (≈75.1% capacitance retention and ≈100% Coulombic efficiency) exceeding the performances of the length‐matched asymmetric YSC. With a maximum energy density of ≈12.3 Wh kg^−1^ (≈154.5 μWh cm^−2^) and power density of ≈650 W kg^−1^ (≈8146.7 μW cm^−2^), the capacitance‐matched asymmetric YSC rivaled the existing asymmetric YSCs. We further used a novel riveted interconnection and carefully integrated three serially connected asymmetric YSCs into a jute fabric. This design allowed for the connection and disconnection of the energy storage fabric as needed (e.g., for washing), establishing charging tabs, and using a protective textile cover layer enabling practical applications of textile‐based power supplies. With a voltage of 3.9 V, this energy‐storing fabric successfully powered a digital clock and a red LED, demonstrating the potential of the asymmetric YSCs for powering a wide range of wearable and portable devices, such as digital watches, health trackers, wireless sensors, and IoT devices. The key advantages of our riveted interconnection strategy include cost‐effectiveness, ease of integration, and compatibility with existing textile manufacturing technology, allowing for the seamless integration of a wide range of YSCs into existing textiles with minimal infrastructure adjustments, opening new avenues for the widespread adoption of YSCs for wearable applications.

## 4 Experimental Section

4.1

4.1.1

##### Materials

MAX (Ti_3_AlC_2_, 98%) phase powder with the particle sizes of <40 μm was purchased from Carbon‐Ukraine Ltd. Hydrochloric acid (HCl, 37%, ACS reagent), potassium hydroxide (KOH, ≥85%, pellets), sodium hydroxide (NaOH, ≥97.0%, pellets), hydrazine hydrate solution (puriss. p.a., 24–26% in H_2_O), and H_2_SO_4_ (95.0‐98.0%) were purchased from Sigma‐Aldrich. Lithium fluoride (LiF, ≥98.5%, ≈325 mesh powder), thioacetamide (98%), DMSO (≥99.5), and sodium molybdate dihydrate (≥99%) were supplied from Thermo Fisher Scientific. PVA (hydrolyzed, molecular weight ≈146 000–186 000, 98%–98.8%) was purchased from Acros Organics. Cotton yarn (linear density of ≈150 tex and diameter of 0.5–0.6 mm) was purchased from Dollfus‐Mieg et Compagnie (DMC) Ltd. Jute fabrics for textile integration were purchased from Neotrims. All chemicals were used as received and without additional purification. Deionized (DI) water (≈0.055 μS cm^−1^) was used in all experiments.

##### Ti_*3*_ *C*_*2*_ *T*_*x*_ *MXene Synthesis*

Ti_3_C_2_T_*x*_ MXene was synthesized using a MILD approach involving a mixture of HCl and LiF as the etchant for the removal of the Al layer from the Ti_3_AlC_2_ MAX phase. First, an etching solution was prepared by mixing HCl (9.13 M) and LiF (1.93 M). The Ti_3_AlC_2_ MAX phase powder (2 g) was then added to the solution and the mixture was kept stirring at 25 °C for 24 h. After etching, the mixture was thoroughly washed using centrifugation (Centurion Scientific Pro Research K241R Centrifuge) at 3 500 rpm (≈1 370 g) for 5 min to remove unreacted materials and by‐products followed by a subsequent centrifugation at 1 500 rpm (≈251.5 g) rpm for 10 min to achieve a stable aqueous dispersion of highly delaminated Ti_3_C_2_T_*x*_ MXene flakes. The concentration of the Ti_3_C_2_T_*x*_ MXene dispersion was then adjusted to the desired range (≈30 mg mL^−1^) using a high‐speed centrifugation step at 10 000 rpm (≈11 190 *g*) for 60 min.

##### rGO/MoS_*2*_ *Synthesis*

GO was synthesized using an improved Hummers’ method.^[^ ^64^ ^]^ An aqueous dispersion of GO (≈5 mg mL^−1^) was used to produce GO/MoS_2_ using a hydrothermal synthesis approach. First, ≈0.96 g of sodium molybdate was added to 80 mL of the aqueous GO solution and bath sonicated (VWR, ultrasonic cleaner, USC‐300 T, 45 kHz) for 20 min. A separate solution was prepared by dissolving ≈0.495 g of thioacetamide in 20 mL of DI water and was mixed with the GO/sodium molybdate mixture by stirring for 20 min. The mixture was then transferred to a Teflon‐lined stainless‐steel autoclave and heated at 185 °C for 20 h in a vacuum oven (Gallenkamp). After the reaction was complete, the mixture was cooled to room temperature. The resulting black precipitate was collected and washed three times using centrifugation at 10 000 rpm (≈11 190 *g*) for 10 min. The as‐synthesized rGO/MoS_2_ (MoS_2_ content of up to ≈67.5 wt%) was then sonicated using a probe sonicator (Branson Sonifier SFX 550, 550 W, power 40%, and pulse 5 s on/off interval) for 30 min, followed by solvent exchange to DMSO using centrifugation at 10 000 rpm (≈11 190 *g*) for 15 min. Two additional rGO/MoS_2_ samples were prepared using ≈0.48 and ≈1.44 g of sodium molybdate along with ≈0.2475 and ≈0.7425 g of thioacetamide, respectively, to achieve varying MoS_2_ contents to identify an optimal composition for electrochemical properties. The MoS_2_ content in rGO/MoS_2_ samples was then estimated using thermogravimetric analysis (Figure S23, Supporting Information).

##### Synthesis of h‐BN Nanosheets

An alkali‐assisted hydrothermal exfoliation process was used for the synthesis of h‐BN nanosheets.^[^ ^65^ ^]^ Briefly, ≈1 g of BN powder was gradually added to ≈50 mL of an alkali solution (≈1.85 M KOH and ≈2.4 M NaOH) while stirring continuously to form a uniform suspension. The resulting mixture was then transferred to a Teflon‐lined autoclave, which was securely sealed and then heated at 185 °C for 20 h in an oven in air. After the reaction was complete, the autoclave was allowed to cool down to the room temperature and the product was washed with DI water using repeated centrifugation at 10 000 rpm (≈11 190 g) for 20 min until the supernatant reached the neutral pH.

##### Preparation of Ti_*3*_ *C*_*2*_ *T*_*x*_ *MXene‐Coated Cotton Yarns and rGO/MoS*_*2*_ *‐Coated Cotton Yarns*

Cotton yarns were used as a sustainable substrate for the preparation of the electrode yarns for YSCs. The Ti_3_C_2_T_*x*_ MXene‐coated cotton yarns were prepared by repeatedly dipping (5–19 times) the cotton yarns in a bath of an aqueous Ti_3_C_2_T_*x*_ MXene dispersion (≈25–30 mg mL^−1^) until the desired mass loading (25–75 wt%) was achieved. A similar approach was used to prepare the rGO/MoS_2_‐coated cotton yarns. The cotton yarns were repeatedly dipped (9–17 times) in a bath containing the rGO/MoS_2_ dispersion (≈−25–30 mg mL^−1^) in DMSO to produce coated yarn with the desired rGO/MoS_2_ mass loading (40–70 wt%). The rGO/MoS_2_‐coated cotton yarn was subsequently treated with an aqueous hydrazine hydrate solution (1:40 ratio) at 80 °C for 6 h to achieve further rGO reduction and was thoroughly washed with DI water to remove excess hydrazine hydrate. The mass loading of Ti_3_C_2_T_*x*_ MXene or rGO/MoS_2_ in the coated yarns was then determined by weighing the yarn before and after the dip‐coating process using a microbalance (Kern, ABT 100‐5NM). To account for the variation in mass along the length of the yarn, the measurements were averaged across three skeins (≈6 cm long). The Ti_3_C_2_T_*x*_ MXene‐ and rGO/MoS_2_‐coated yarns were thoroughly air‐dried and then placed in a vacuum desiccator to ensure complete drying and to minimize MXene or MoS_2_ oxidative degradation before further evaluation.

##### Characterizations

The XRD spectra of the Ti_3_C_2_T_*x*_ MXene, rGO/MoS_2_, pristine cotton, and Ti_3_C_2_T_*x*_ MXene‐ and rGO/MoS_2_‐coated cotton yarns were obtained using an X‐ray diffractometer (Bruker D2 PHASER) equipped with a Cu Kα radiation source (λ = 0.1542 nm) and a graphite Kβ filter. The spectra were recorded at a voltage of 40 kV and a current of 15 mA, covering a 2*θ* range of 4°–80° with a step size of 0.04°. The Raman spectra of the Ti_3_C_2_T_*x*_ MXene and rGO/MoS_2_ powder samples were obtained using a confocal Raman microscope (Horiba LabRAM HR Evolution) equipped with a 532 nm excitation laser. Surface characterization of the sample was carried out using an XPS (PHI VersaProbe 5000) with a 200 μm and 50 W monochromatic Al‐Kα (1,486.6 eV) X‐ray source. The optoelectronic properties of the Ti_3_C_2_T_*x*_ MXene and rGO/MoS_2_ dispersions were investigated by recording the UV–Vis (Agilent Cary 60) absorption spectra. The hydrodynamic diameter and zeta potential of the Ti_3_C_2_T_*x*_ MXene and rGO/MoS_2_ dispersions were measured using DLS (Malvern Zetasizer Nano ZS) with a 632.8 nm laser and a scattering angle of 173°. The morphological observation and elemental analysis of the samples were carried out using an SEM (Zeiss Auriga) equipped with an EDS detector (Oxford Instruments). High‐resolution nanostructural images of the materials were obtained using an HRTEM (JEOL JEM‐2100 F). The samples for TEM were prepared by depositing the Ti_3_C_2_T_*x*_ MXene or rGO/MoS_2_ flakes on a carbon coated copper grid via drop‐casting. The electrical conductivity of the Ti_3_C_2_T_*x*_ MXene‐ and rGO/MoS_2_‐coated cotton yarns was evaluated using a custom‐designed four‐point probe cell with the aid of a source meter unit (Keysight B2901A). The electrical conductivity of Ti_3_C_2_T_*x*_ MXene and rGO/MoS_2_ films was measured using a four‐point probe conductivity meter (Jandel RM3000+).

##### Preparation of Gel Electrolyte

A PVA gel was initially prepared by dissolving PVA (≈2 g) in DI water (≈18.9 mL) at 85 °C for 8 h under vigorous stirring. After cooling, H_2_SO_4_ (≈1.122 mL, 95% w/w) was added to the PVA solution, and the mixture was kept stirring at room temperature for 1 h to achieve the PVA/H_2_SO_4_ gel electrolyte. The PVA–H_2_SO_4_/h‐BN gel electrolyte was then prepared by adding h‐BN nanosheets (≈0.15 g) to the PVA/H_2_SO_4_ gel electrolyte and stirring for 1 h. The gel electrolytes were kept under vacuum for 4 h to remove the bubbles before use.

##### Fabrication of the Symmetric and Asymmetric YSCs

The symmetric YSCs were developed by using two electrodes of the same kind, that is, either the Ti_3_C_2_T_*x*_ MXene‐ or rGO/MoS_2_‐coated cotton yarn, at the same length and placing them next to each other. The electrode yarns and the space in between were then covered by a layer of the gel electrolyte. The YSC was then kept at the room temperature overnight to ensure complete wetting and evaporation of excess water. The asymmetric YSCs were prepared using a similar approach but with two different electrodes, that is, the Ti_3_C_2_T_*x*_ MXene‐coated yarn as the negative electrode and the rGO/MoS_2_‐coated yarn as the positive electrode. The lengths of the electrode yarns were adjusted to achieve asymmetric YSCs with matching length, matching capacitance, or matching both length and capacitance.

##### Textile Integration of Asymmetric YSCs and Interconnections

The Ti_3_C_2_T_*x*_ MXene‐coated cotton yarn negative electrode and the rGO/MoS_2_‐coated cotton yarn positive electrode were first coated with a layer of the gel electrolyte, and then, carefully stitched into a piece of jute fabric in order to achieve asymmetric YSCs. The negative and positive electrode yarns were placed parallel to each other and in very close proximity, leaving only one weft yarn of the host fabric as a separator. A layer of the gel electrolyte was then applied to the electrodes to cover the surface and the space between the electrodes. Three asymmetric YSCs were integrated in the fabric using this approach to allow for the serial or parallel interconnections as necessary. Each yarn electrode was then connected to a conductive male snap rivet, enabling the interconnection of the asymmetric YSCs to establish various circuits (i.e., serial or parallel) depending on the arrangement of the rivets. Two female snap rivets were also used on a different section of the host fabric and were connected to two pieces of wires to establish charging tabs and allow for the interconnected asymmetric YSCs to be used for powering electronic devices.

##### Electrochemical Testing of the Yarn Electrodes and YSCs

The electrochemical properties of the Ti_3_C_2_T_*x*_ MXene‐ and rGO/MoS_2_‐coated cotton yarn electrodes (length ≈1.5–1.8 cm) were investigated in a three‐electrode system in H_2_SO_4_ (1 M) electrolyte using a Pt mesh (1 × 1 cm) as the counter electrode and Ag/AgCl (NaCl 3 M) as the reference electrode. A two‐electrode system was used for the symmetric and asymmetric YSCs. The electrochemical testing of the yarn electrodes and YSCs was carried out using an electrochemical workstation (BioLogic SP‐300) with the CV, GCD, and EIS measurements. The CV and GCD curves were recorded at various scan rates (2–100 mV s^−1^) and current densities (0.05–3 A g^−1^), respectively. The EIS measurements were conducted at the open‐circuit potential by applying a sinusoidal potential signal with an amplitude of 10 mV in a frequency range of 10 mHz to 1 MHz. The capacitance was calculated based on the CV and GCD results using Equation (1) and (2), respectively.

(1) $$C=\;\frac{\int (\text{I}V)\text{d}V}{2s\Delta V}$$

(2) $$C=\frac{I\Delta t}{\Delta V}$$

Here, *C* is capacitance (F), *I* is current (A), *ΔV* is potential or voltage (V), *s* is scan rate (V s^−1^), and *t* is discharge time (s), respectively. The specific capacitances (*C*_s_) of the electrode including gravimetric capacitance (*C*_G_, F g^−1^), areal capacitance (*C*_A_, mF cm^−2^), length capacitance (*C*_L_, mF cm^−1^), or volumetric capacitance (*C*_V_, mF cm^−3^) were calculated by normalizing the capacitance to the active mass, surface area, length, or volume of the yarn electrode, respectively. For YSCs, the specific capacitances (*C*_s_) were determined by normalizing the capacitance to the total mass of the active materials, surface area, length, and volume of the entire device, respectively. The energy density (*E,* Wh kg^−1^ or μWh cm^−2^) and the power density (*P,* W kg^−1^ or μW cm^−2^) of the YSCs were calculated using Equation (3) and (4), respectively.

(3) $$E=\frac{1}{2}C_{s}V^{2}$$

(4) $$P=\;\frac{E}{t}$$

The washability of asymmetric YSC was evaluated by comparing the CV behaviors at a scan rate of 2 mV s^−1^ before and after two washing cycles. To simulate a realistic washing scenario, the YSC was immersed in 150 mL of tap water containing 200 μL of a household laundry detergent and stirred continuously at 250 rpm for 15 min. After each washing cycle, any residual laundry detergent was thoroughly rinsed off with tap water. The YSC was then dried on a hot plate at 35 °C for 2 h and the electrolyte was replenished before the CV measurement.

## Conflict of Interest

The authors declare no conflict of interest.

## Supporting information

Supplementary Material

## Data Availability

The data that support the findings of this study are available from the corresponding author upon reasonable request.
