# Supplementary material for: Riveted Interconnections of Capacitance‐Matched MXene‐Based Yarn Supercapacitors Enable Seamless Energy Integration in Textiles
Source: Small Sci. 2025 Jul 6;5(9):2500229. doi: 10.1002/smsc.202500229 (PMC12412493; doi:10.1002/smsc.202500229)
Supplement: Supplementary file 1 — Supplementary Material [file SMSC-5-2500229-s001.zip › Smsc.202500229-sup-0001-suppdata-S1.pdf]

Supporting Information

Riveted Interconnections of Capacitance-Matched MXene-Based Yarn Supercapacitors Enable Seamless Energy Integration in Textiles

*Neeraj Kumar, Patryk Wojciak, and Shayan Seyedin**

N. Kumar, P. Wojciak, and S. Seyedin*

School of Engineering, Newcastle University, Newcastle upon Tyne, NE1 7RU, United Kingdom

E-mail: Shayan.Seyedin@newcastle.ac.uk

Characterization of h-BN nanosheets

Hexagonal boron nitride (h-BN) is a two-dimensional (2D) material composed of boron and nitrogen atoms arranged in a hexagonal lattice. h-BN is known for its large band gap, excellent insulating properties, high thermal conductivity, and chemical stability. We synthesized h-BN nanosheets using an alkali-assisted hydrothermal approach and characterized by XRD, SEM, and TEM. The XRD pattern of h-BN nanosheets (**Figure S1a**) showed diffraction peaks at 26.4°, 41.5°, 43.6°, 54.9°, and 75.8°, corresponding to the (002), (100), (101), (004), and (110) planes, respectively, confirming the successful formation of h-BN. SEM (**Figure S1b**) and TEM (**Figure S1c**) analyses confirm that h-BN exhibits a hexagonal crystalline structure, with a diameter ranging from ≈100-250 nm and a thickness between ≈10-40 nm.

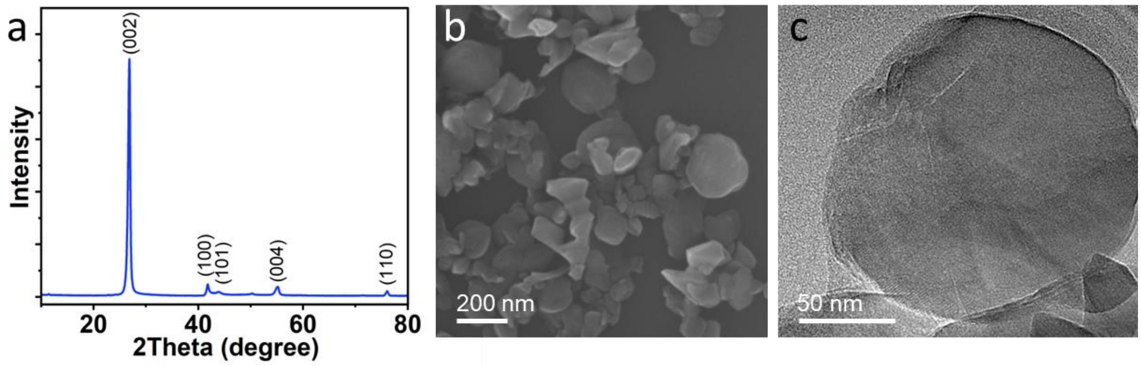

Figure S1. (a) XRD, (b) SEM image, and (c) TEM image of h-BN nanosheets.

Raman analysis of $Ti_3C_2T_x$ MXene and rGO/MoS_2

The Raman spectrum of $Ti_3C_2T_x$ MXene features two key spectral regions (**Figure S2a**): the low-frequency region (100-800 cm^{-1}), representing lattice vibrations or phonons, and the mid-frequency region (1000-1800 cm^{-1}), associated with C−C stretching vibrations within the carbon structures present in MXene. The peaks at 118 (titanium E_g mode) and 196 cm^{-1} (titanium $A1_g$ mode) are associated with the multiple different MXene types present in the sample.[1] The appearance of the peak at 371 cm^{-1} corresponds to the in-plane vibrations of planar Ti–C in the $Ti_3C_2T_x$ MXene.[1a] Raman bands at 255 and 448 cm^{-1} are related to surface groups ($Ti_3C_2F_2$ and $Ti_3C_2(OH)_2$) of the MXene, respectively. The band at 584 cm^{-1} is assigned to $Ti_3C_2(O)_2$ MXene structures. The complex peak at 628 cm^{-1} is associated with the out-of-plane vibration of the Ti-C within the $Ti_3C_2T_x$ MXene structure.[2] Furthermore, Raman-active

modes in the disordered carbon spectral range of 1200–1700 cm^{-1}, corresponding to the D and G bands of graphitic carbon, suggest structural distortions in the $Ti_3C_2T_x$ MXene. Raman spectrum of rGO/MoS_2 (**Figure S2b**) exhibits two characteristic peaks of hexagonal-MoS_2 in the range 365-406 cm^{-1}. These bands (403 and 370 cm^{-1}) correspond to the out-of-plane (^1A_g) vibrational modes of sulfur atoms and the in-plane ($^1E_{2g}$) vibrations of sulfur atoms on molybdenum, respectively. Furthermore, the higher intensities of ^1A_g compared to $^1E_{2g}$ vibrational modes suggest the development of edge-terminated 2H MoS_2 nanosheets on rGO.[3] The E_{1g} mode near 285 cm^{-1} also appears in 2H MoS_2 because it lies at the Γ point of the hexagonal Brillouin zone.[4] Although this mode is forbidden in backscattering experiments on surfaces perpendicular to the *c* c-axis, its observation suggests that the MoS_2 layers were randomly oriented, which eliminates any polarization effects.[4b] The distinct peaks in the carbon spectral region at 1352 and 1588 cm^{-1} correspond to the D and G bands, respectively. The D band arises from disorder and defects in the graphite lattice due to disruptions in the sp^2 hybridized carbon, while the G band is associated with the in-plane vibrations of sp^2 hybridized carbon atoms, represents graphitic carbon and first-order scattering of the E_{2g} phonon.

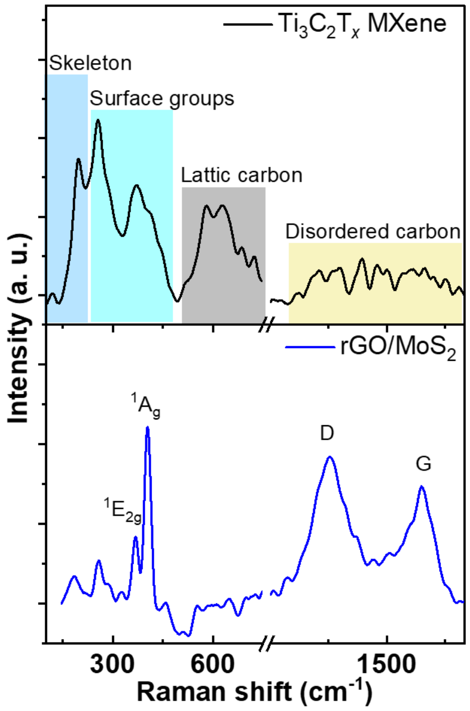

Figure S2. Raman spectra of (a) $Ti_3C_2T_x$ MXene and (b) rGO/MoS_2.

XPS studies of $Ti_3C_2T_x$ MXene and rGO/MoS_2

XPS was used to examine the bonding behaviour of constituent elements in $Ti_3C_2T_x$ MXene and rGO/MoS_2 and to detect all elements, including those present in low concentrations or confined to edges and surfaces. **Figures S3a-d** show the fitted XPS spectra of constituent elements of $Ti_3C_2T_x$ flakes. The Ti 2p spectrum exhibits doublets at ≈455 and ≈462 eV, corresponding to Ti $2p_{3/2}$ and Ti $2p_{1/2}$, respectively, originating from spin-orbit splitting.[5] Upon applying component peak fitting to deconvolute these peaks, it was noticed that all components that align with Ti $2p_{3/2}$ peak were also present under the Ti $2p_{1/2}$ peak. Four components were fit to Ti $2p_{3/2}$ spectra at 454.93 ($\Delta_{Ti2p1/2}$ = 5.97 eV), 456.15 ($_{Ti2p1/2}$ = 5.97 eV), 457.65 ($_{Ti2p1/2}$ = 5.21 eV) and 459.2 eV ($_{Ti2p1/2}$ = 5.08 eV) corresponding to C-Ti-C, C-Ti-O, C-Ti-F and $TiO_{2-x}F_{2x}$, respectively.[6] The first peak at 454.91 eV was attributed to Ti bonded exclusively to C, while the second (456.13 eV), third (457.70 eV), and fourth peaks (459.24 eV) were assigned to Ti atoms bonded to pure O, pure F terminations, and a mix of O and F, respectively. In the C 1s spectra, three components were fitted at 281.98, 284.51 and 285.7 eV, respectively. The first peak (281.98 eV) was assigned to $Ti_3C_2T_x$ MXene carbon (Ti–C–Ti), while the other two peaks were attributed to adventitious carbon contaminants. The O 1s spectra were fitted with three components related to C–Ti–O, C-Ti-OH, and adsorbed H_2O at 531.7, 532.6, and 532.2 eV, respectively. Similarly, the F 1s spectra revealed two peaks at 685.2 eV and 686.7 eV, corresponding to C–Ti–F and fluorine contaminants (e.g., AlF_x).

The high-resolution spectra of all elements (Mo, S, C, and O) of rGO/MoS_2 are shown in **Figure S3e-h**. The Mo 3d spectrum of MoS_2 displays well-resolved doublet peaks at 228.6 eV ($3d_{5/2}$) and 231.9 eV ($3d_{3/2}$), indicating the presence of Mo(IV) oxidation state.[7] The weak peak at 225.8 eV was attributed to S 2s. Additionally, two additional peaks at 235.5 eV ($3d_{3/2}$) and 233.7 eV ($3d_{5/2}$) are attributed to the Mo(VI) oxidation state. The S 2p spectrum is notably broadened and could be deconvoluted into two doublets at 161.4 eV ($2p_{3/2}$) and 162.8 eV ($2p_{1/2}$), corresponding to sulfur associated with the 2H phase of MoS_2. An additional peak at 168.5 eV is attributed to the presence of sulfate groups.[8] The C1s spectrum of rGO/MoS_2 could be deconvoluted into four component peaks at 284.4, 285.5, 286.5, and 288.9 eV, corresponding to C=C/C-C, C-O, C=O, and COOH groups of rGO. Furthermore, **Figure S3h** presents the singlet O 1s spectrum, featuring component peaks at 533.8 eV, 532.8 eV and 531.0 eV, which are assigned to C=O, C–OH/C–O–C, and O=C–O groups, respectively.

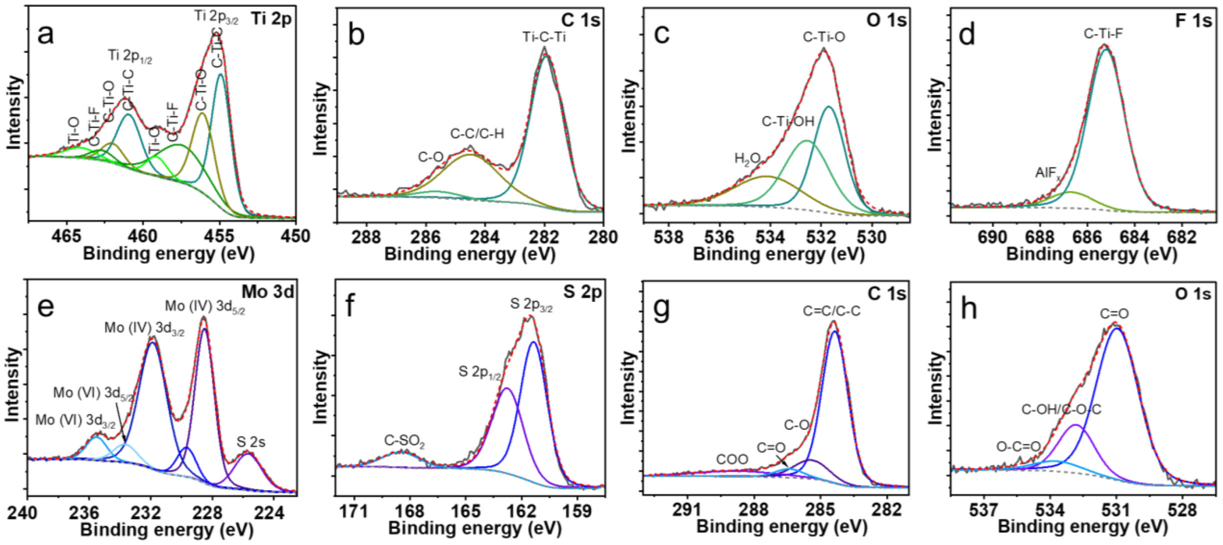

Figure S3. XPS deconvoluted spectra of $Ti_3C_2T_x$ MXene for (a) Ti 2p, (b) C1s, (c) O 1s, and (d) F 1s. XPS deconvoluted spectra of rGO/MoS_2 for (e) Mo 3d, (f) S 2p, (g) C 1s, and (h) O 1s.

Optical and morphological characterization of $Ti_3C_2T_x$ MXene and rGO/MoS_2

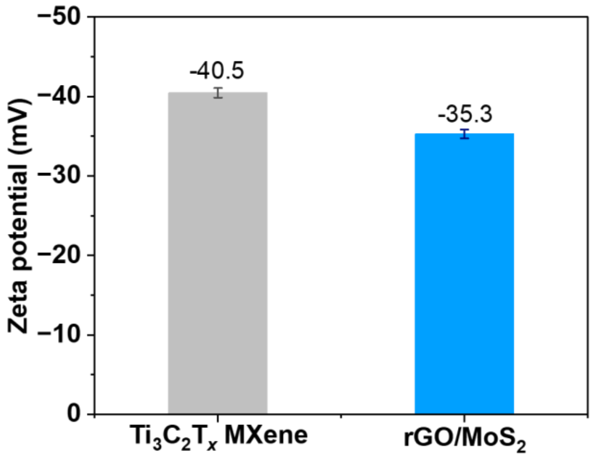

Figure S4. Zeta potential of $Ti_3C_2T_x$ MXene and rGO/MoS_2.

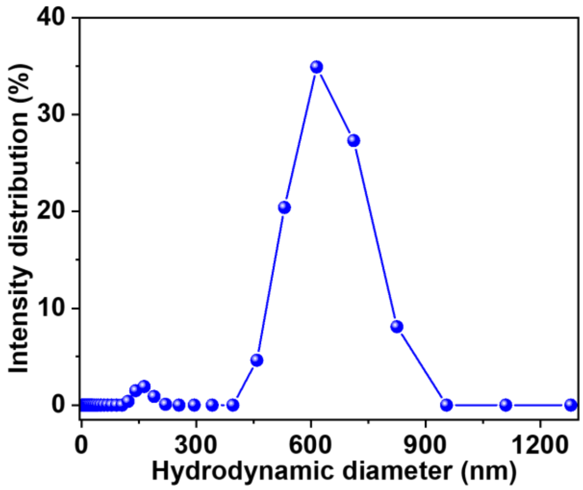

Figure S5. DLS size distribution of $Ti_3C_2T_x$ MXene flakes.

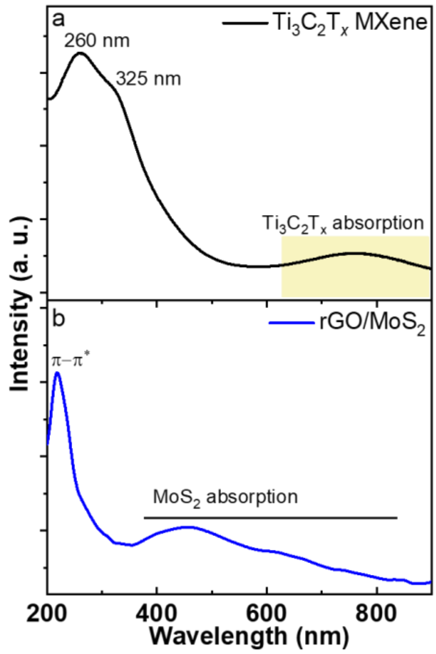

Figure S6. UV-Vis spectra of (a) $Ti_3C_2T_x$ MXene and (b) rGO/MoS_2.

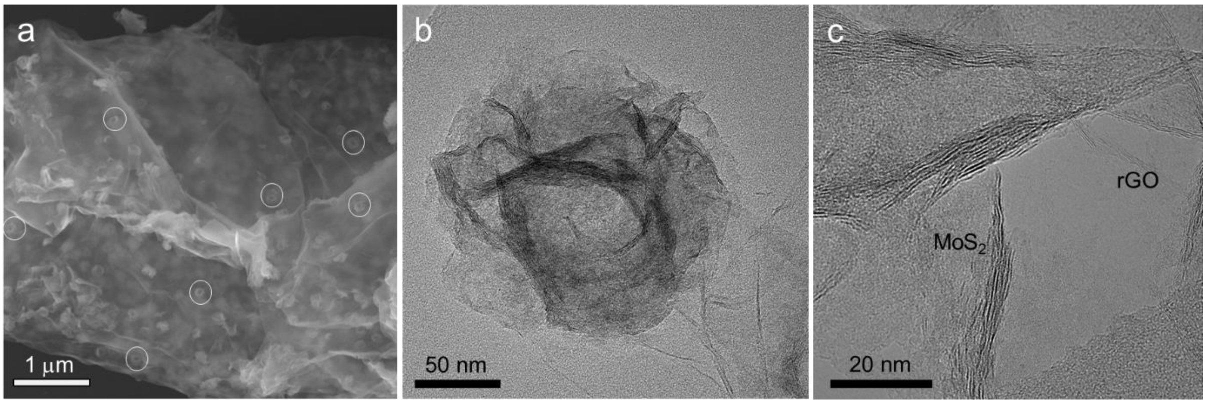

Figure S7. (a) SEM image of rGO/MoS_2 showing distinct hollow MoS_2 nanoflowers highlighted by circles, (b) TEM image of rGO/MoS_2 showing dark boundaries and a light hollow center in a typical MoS_2 nanoflower, and (c) high-resolution TEM image of rGO/MoS_2 showing the presence of the rGO substrate and MoS_2 nanoflowers indicated by visible lattice fringes of MoS_2.

Chemical, structural and morphological characterization of $Ti_3C_2T_x$ MXene- and rGO/MoS_2-coated cotton yarns

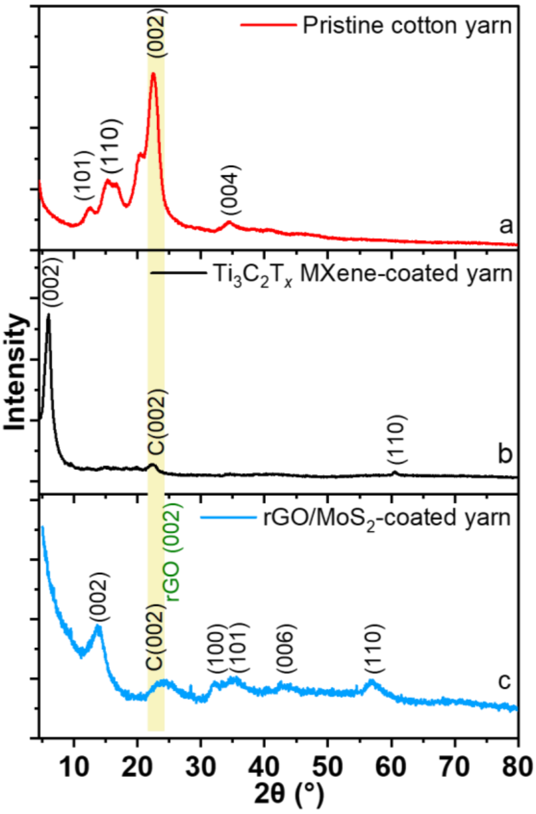

Figure S8. XRD spectra of pristine cotton (a), $Ti_3C_2T_x$ MXene-coated cotton yarn (b), and rGO/MoS_2-coated cotton yarn (c).

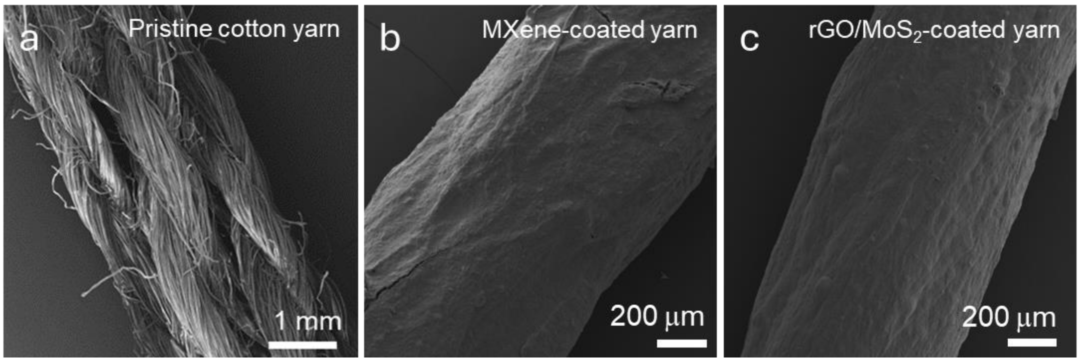

Figure S9. SEM image of (a) uncoated cotton yarn revealing multiple filaments, (b) MXene-coated yarn, and (c) rGO/MoS_2-coated cotton yarn.

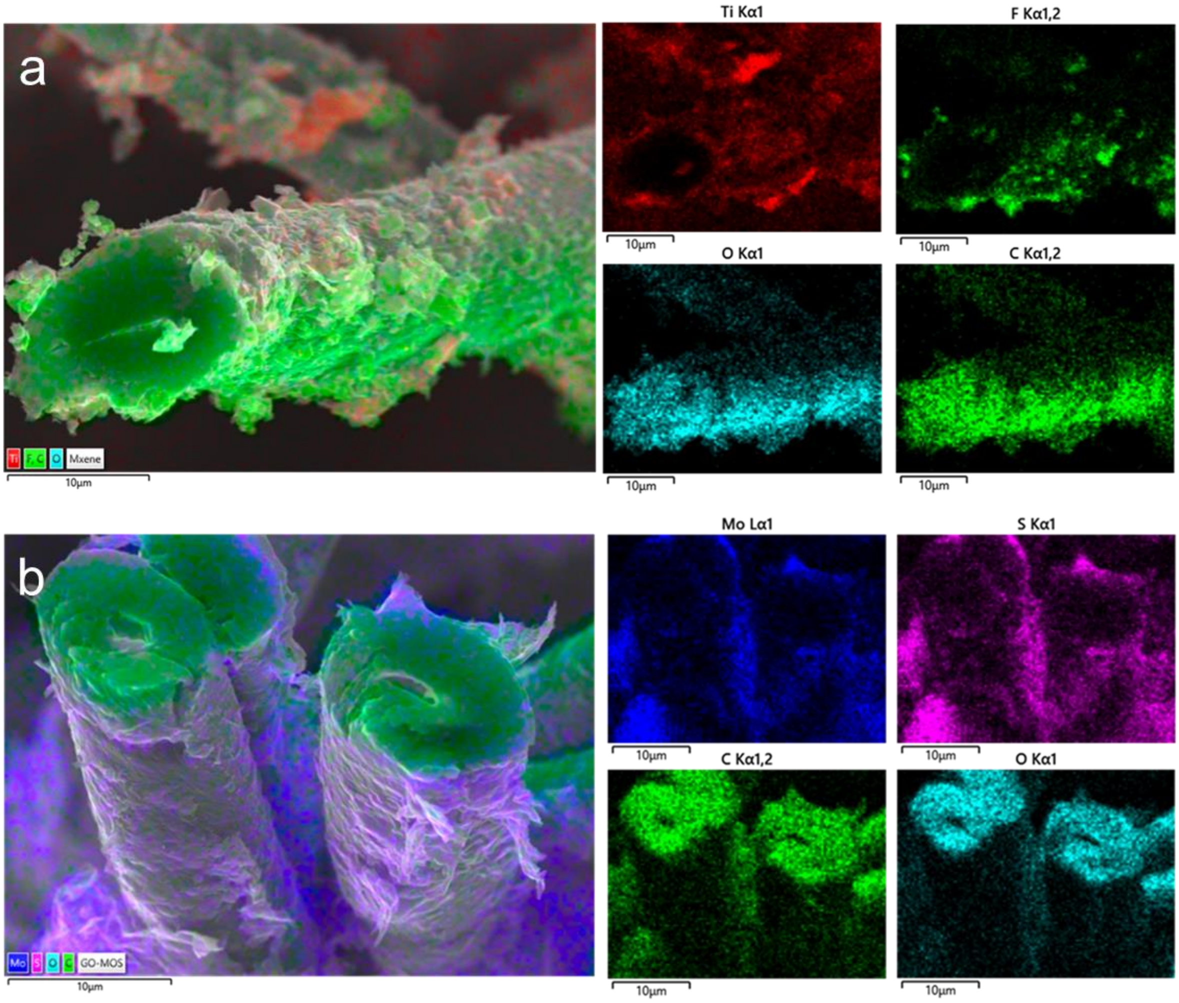

Figure S10. (a) EDS of a typical $Ti_3C_2T_x$ MXene-coated cotton yarn (coating of 45 wt%) showing the presence of Ti, C, F, and O, confirming the successful coating of MXene. **(b)** EDS of a typical rGO/MoS_2-coated cotton yarn (coating of 50 wt%, ≈67.5 wt% MoS_2) showing the presence of Mo, S, C, and O, confirming the successful coating of rGO/MoS_2.

Mechanical properties of $Ti_3C_2T_x$ MXene- and rGO/MoS_2-coated cotton yarns

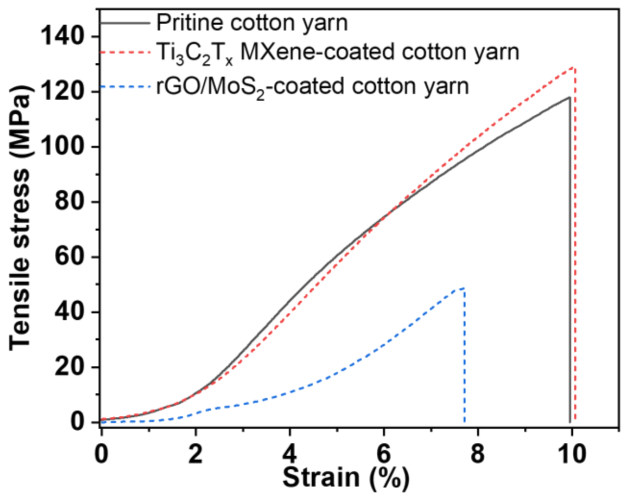

Figure S11. Tensile stress-strain curves of pristine cotton yarn, $Ti_3C_2T_x$ MXene-coated cotton yarns (45 wt.%), and rGO/MoS_2-coated cotton yarns (50 wt.% yarn mass loading with ≈67.5 wt.% MoS_2).

Electrical conductivities of $Ti_3C_2T_x$ MXene- and rGO/MoS_2-coated cotton yarns

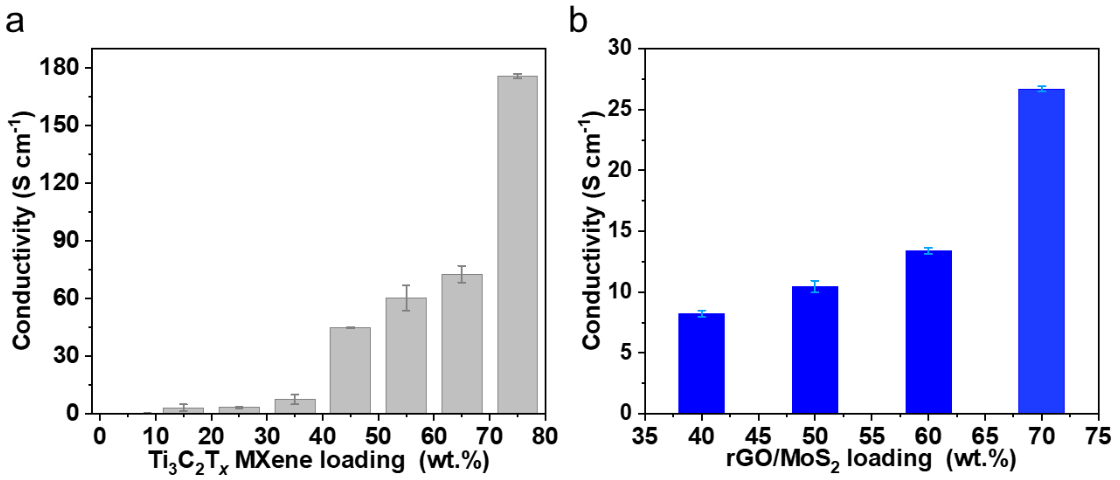

Figure S12. Electrical conductivities of (a) $Ti_3C_2T_x$ MXene-coated cotton yarns and (b) rGO/MoS_2-coated cotton yarns at various mass loadings.

Electrochemical characterization of $Ti_3C_2T_x$ MXene- and rGO/MoS_2-coated cotton yarns

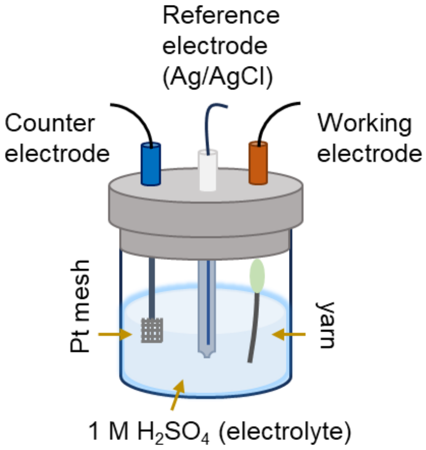

Figure S13. Schematic illustration of the three-electrode setup used for evaluating the electrochemical performance of the electrode yarns.

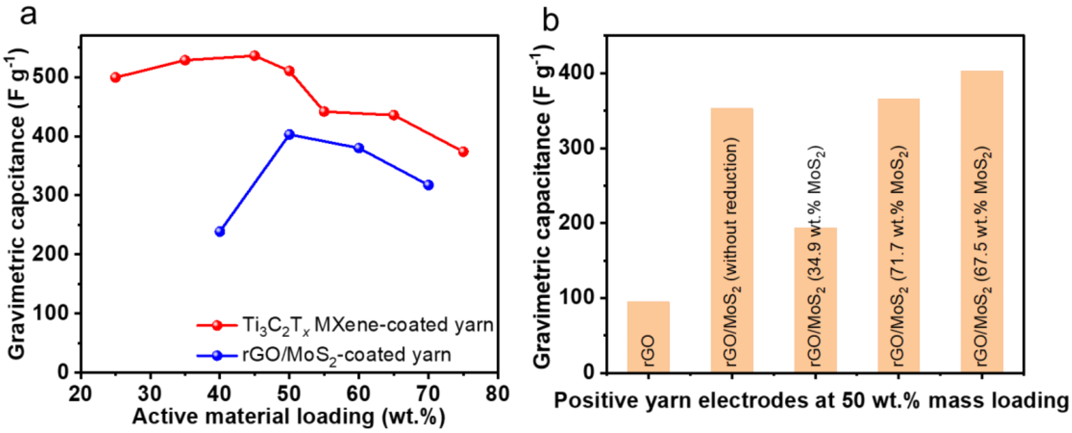

Figure S14. (a) Gravimetric capacitance of $Ti_3C_2T_x$ MXene-coated cotton yarns and rGO/MoS_2-coated yarn with different loading of active materials. (b) Gravimetric capacitance of various positive yarn electrodes at 50 wt% mass loading.

CV measurements were used at various scan rates (1–50 mV s^{-1}) to investigate the electrochemical reaction kinetics (**Figure S15a and b**). The relationship between the anodic peak current (i) and the scan rate (v) was analyzed using the power-law equation, as described in Equation (S1).

$$i = av^b \quad \text{(S1)}$$

In this context, b represents the slope of the log(i) *vs.* log(v) and a is a constant. The value of b provides valuable insights into the charge storage mechanism. For instance, a b-value of 0.5 indicates a diffusion-controlled process, whereas a b-value of 1.0 suggests a surface-controlled (capacitive) process. For our electrodes, we calculated a b-value of 0.65 for $Ti_3C_2T_x$ MXene-coated cotton yarn (**Figure S15a**) and a b-value of 0.50 for the rGO/MoS_2-coated yarn (**Figure S15b**). These results indicate that the $Ti_3C_2T_x$ MXene-coated electrode exhibited a mixed charge storage mechanism, involving both diffusion-controlled and capacitive contributions, while the rGO/MoS_2-coated yarn electrode followed a predominantly diffusion-controlled process.

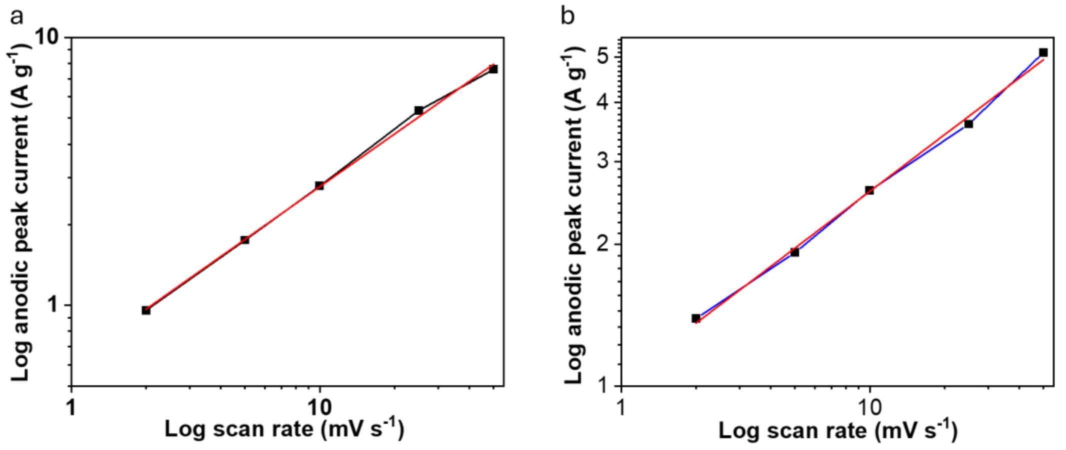

Figure S15. Log anodic peak current vs. log scan rate diagrams (a) $Ti_3C_2T_x$ MXene-coated cotton yarns and (b) rGO/MoS_2-coated yarn.

Symmetric YSCs

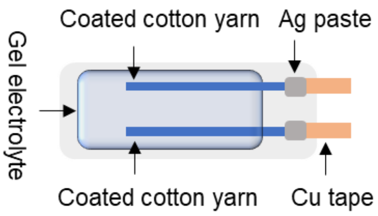

Figure S16. Schematic illustration of symmetric YSC.

We calculated gravimetric capacitances of 85.19 F g^{-1} in PVA-H_2SO_4/h-BN and 80.62 F g^{-1} in PVA-H_2SO_4 electrolyte at a current density of 0.05 A g^{-1} (**Figure S15**). This enhancement in electrochemical properties in PVA-H_2SO_4/h-BN could be attributed to the stabilized electrolyte-electrode interface due to the excellent chemical and thermal stability of h-BN. However, increasing the loading of h-BN in the gel electrolyte negatively impacted the overall electrochemical performance of the coated yarn electrode. This is likely due to increased tortuosity in ion diffusion pathways and higher internal resistance within the system, attributed to the insulating nature of h-BN. The addition of h-BN to the gel electrolyte leveraged its inert nature, enhancing the gel electrolyte resistance to degradation under various environmental conditions and provided long-term reliability while also helping to achieve a higher proximity of the electrodes and prevent short circuits.

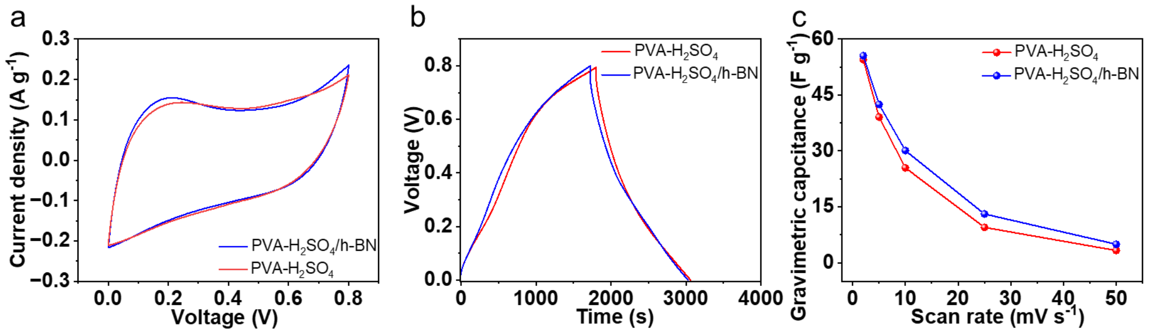

Figure S17. Symmetric YSCs obtained by using the $Ti_3C_2T_x$ MXene-coated yarn electrodes and different electrolytes: (a) CV curves at 2 mV s^{-1}, (b) GCD curves at 0.05 A g^{-1}, and (c) rate capability performances.

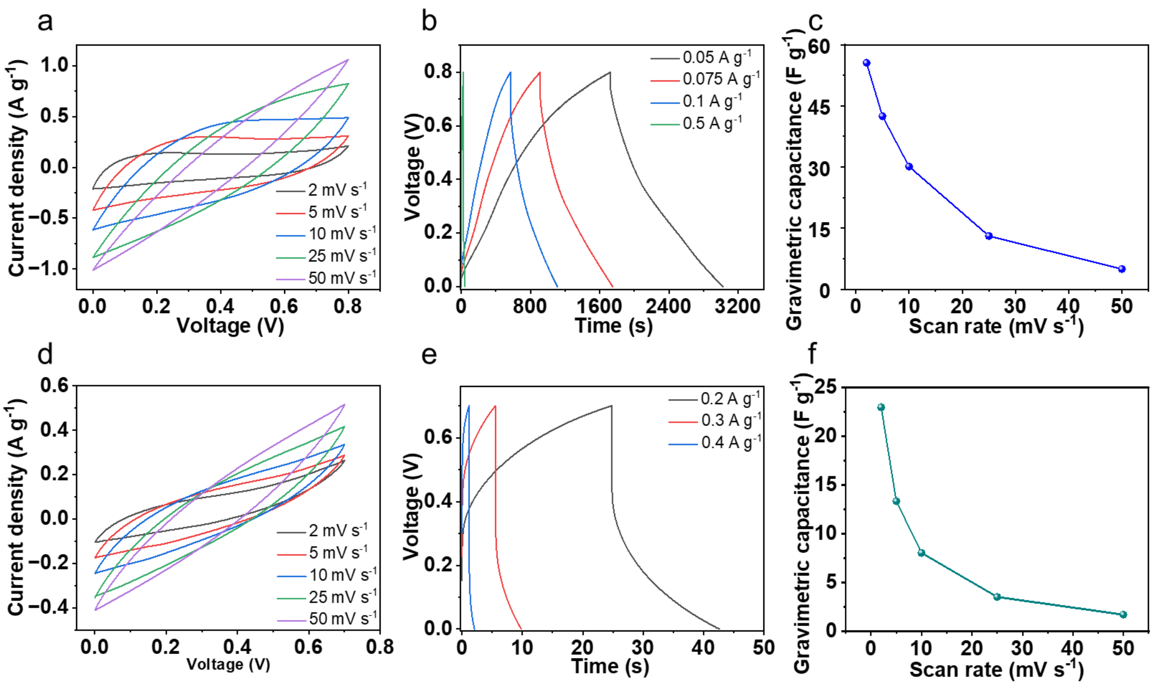

Figure S18. Symmetric YSCs obtained by using the $Ti_3C_2T_x$ MXene-coated yarn electrodes: (a) CV curves, (b) GCD curves, and (c) rate capability performance. Symmetric YSCs obtained by using the rGO/MoS_2-coated yarn electrodes: (d) CV curves, (e) GCD curves, and (f) rate capability performance.

Asymmetric YSCs made using various approaches

Asymmetric supercapacitors are typically constructed using distinct positive and negative electrodes to maximize the operating voltage and energy density. A critical aspect in achieving high performance and maximizing energy output in asymmetric supercapacitors is the maintenance of charge balance between the positive and negative electrodes (Q_+=Q_-). This charge balance is governed by Equation (S2).

$$C_+V_+M_+ = C_-V_-M_- \tag{S2}$$

where $C_{+/-}$, $V_{+/-}$, $M_{+/-}$ denote the specific capacitance, potential window, and mass (volume or length) of the positive and negative electrodes, respectively. Mathematically, the specific capacitance of an asymmetric supercapacitor comprising two different electrodes can be expressed using Equation (S3).

$$C = \frac{Q}{(M_+ + M_-)V} \tag{S3}$$

or

$$C = \frac{Q}{(Q/C_+V_+ + Q/C_-V_-)V} \tag{S4}$$

which can be simplified as

$$\frac{1}{C} = \frac{1}{aC_+} + \frac{1}{bC_-} \tag{S5}$$

where $V_+ = aV$ and $V_- = bV$, with a and b being the proportionality constants related to mass (volume or length) of the electrodes. Hence, the maximum specific capacitance of an asymmetric supercapacitor is achieved when $aC_+ = bC_-$. This indicates that the capacitances of the two electrodes must be equal (matched) to maintain charge balance. As the C and V values of the positive and negative electrodes can be different, charge balance is typically achieved by adjusting the mass ratio between the positive and negative electrodes in conventional planar asymmetric supercapacitors. However, in asymmetric yarn supercapacitors (YSCs), such adjustments are difficult given the differences in active material mass loadings in the positive and negative electrodes.

At a given mass loading, the mass and volume of the yarn electrode are proportional to its length, providing a facile approach to achieving capacitance matching for YSCs. In this manuscript, we proposed to achieve capacitance matching by adjusting the length (L) of the electrodes. This can be mathematically expressed using Equation (6).

$$C_+V_+L_+ = C_-V_-L_- \tag{S6}$$

The optimal length ratio between the positive and the negative electrodes could be easily achieved by simply cutting the electrode yarns with the desired lengths post-production. This strategy is more feasible and controllable than through active material mass adjustment which requires careful control of active material deposition during electrode yarn production.

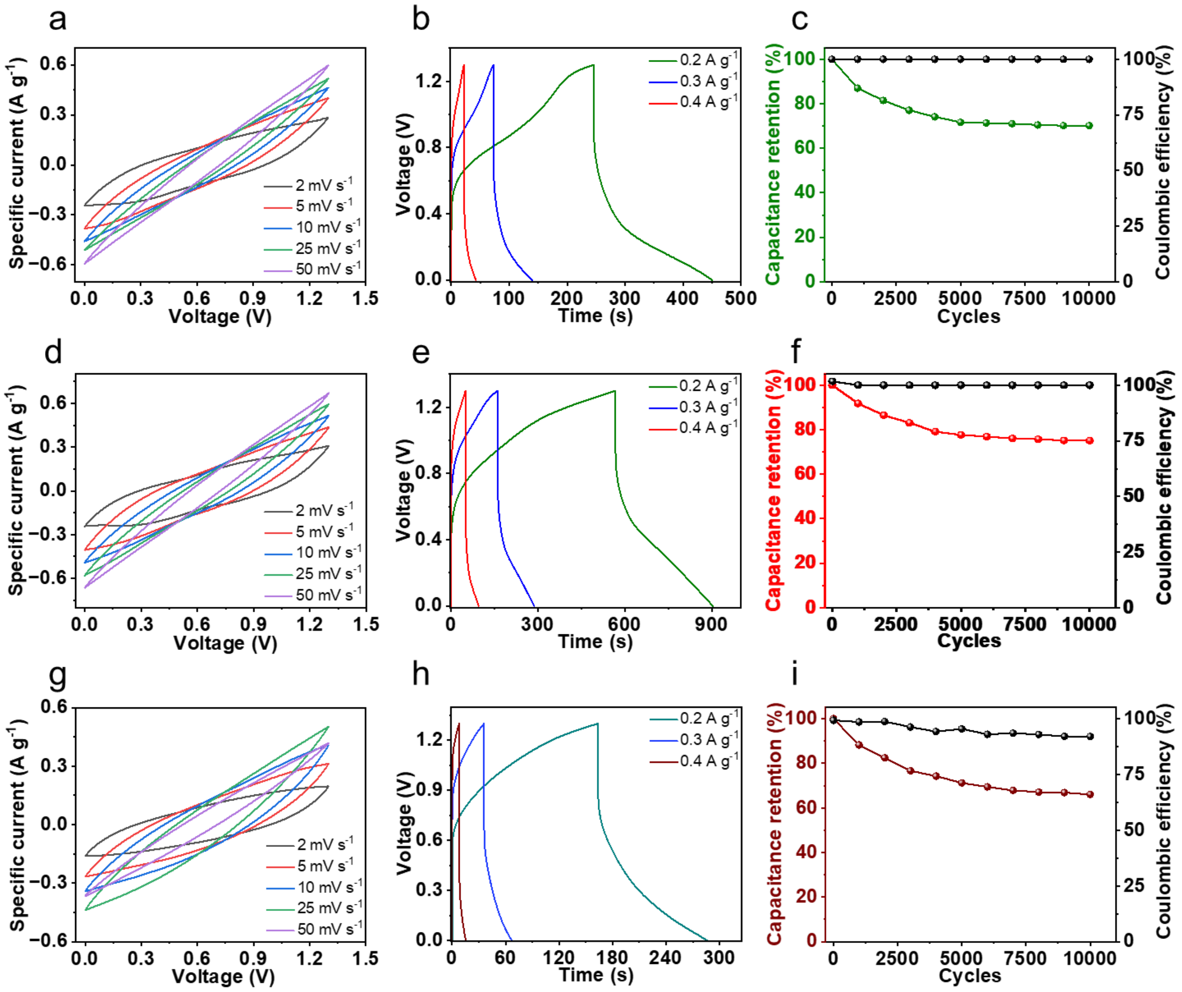

Figure S19. Length-matched asymmetric YSC made using two equal-length electrodes: (a) CV curves, (b) GCD curves, and (c) cyclic stability, including coulombic efficiency. Capacitance-matched asymmetric YSC made using increasing the length of positive electrode: (d) CV curves, (e) GCD curves, and (f) cyclic stability, including coulombic efficiency. Length- and capacitance-matched YSC made using an additional positive electrode yarn: (g) CV curves, (h) GCD curves, and (i) cyclic stability, including coulombic efficiency.

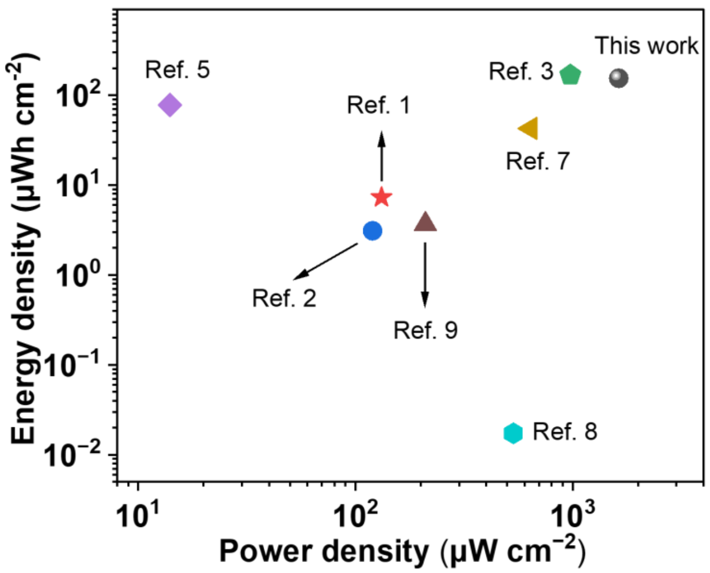

Figure S20. Ragone plot comparing the energy density and power density of the asymmetric YSC developed in this work with the literature results.

Mechanical robustness and washability studies of asymmetric YSCs

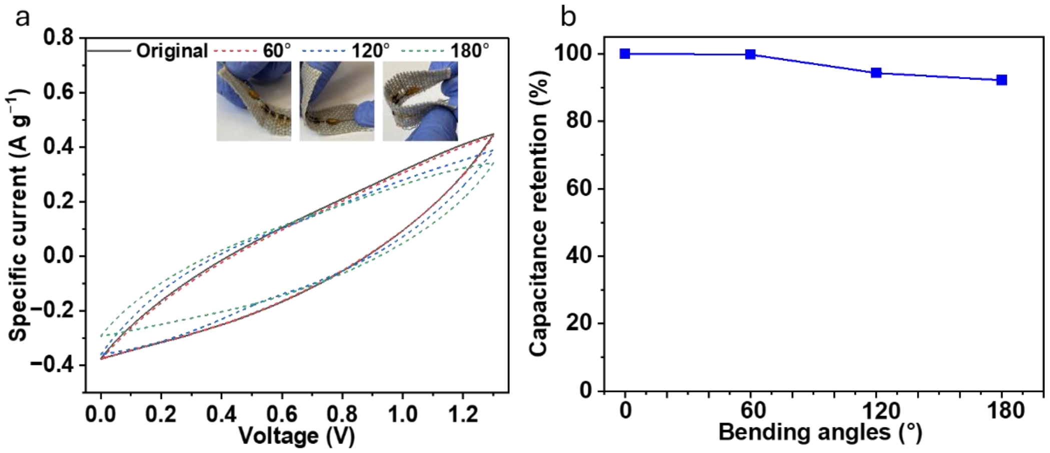

Figure S21. (a) CV of the asymmetric YSC bent to different degrees at 5 mVs^{-1} (inset photographs showing the YSC under different bending angles). (b) Capacitance retention of the asymmetric YSC under different bending angles.

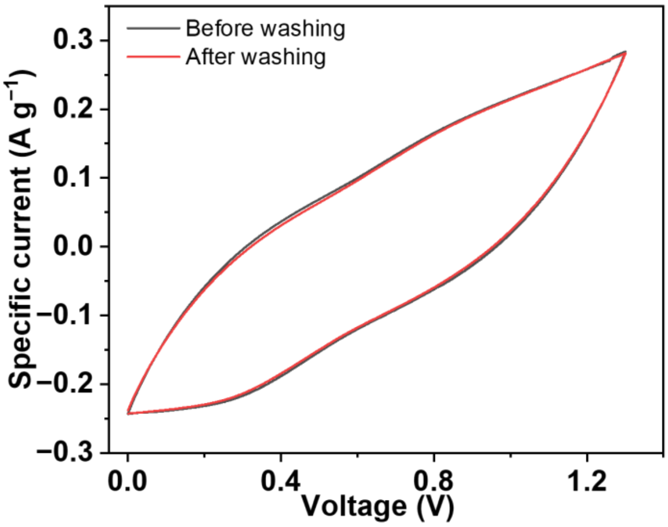

Figure S22. CV curves of the asymmetric YSC before and after two washing and drying cycles.

Thermogravimetric analysis of various rGO/MoS$_2$ samples

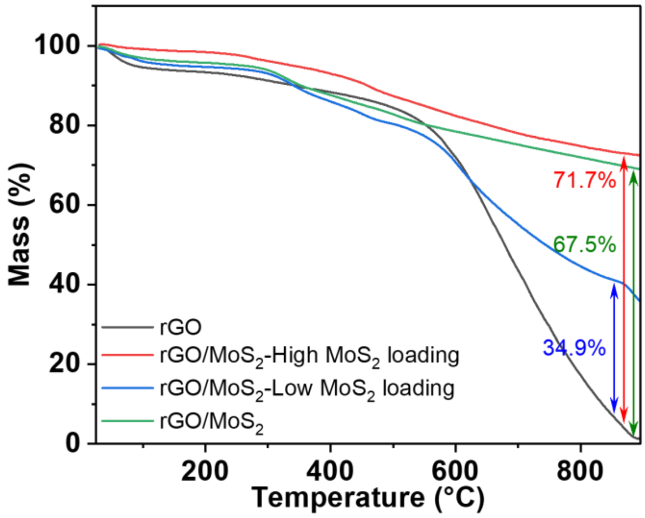

Figure S23. TGA curves of rGO and rGO/MoS$_2$ samples with different MoS$_2$ contents.

Table S1. Comparison of capacitance, specific energy, and power density of various yarn-based supercapacitors

**Material**	**C_A (mF cm^{-2})**	**C_L (mF cm^{-1})**	**C_G (F g^{-1})**	**C_V (F cm^{-3})**	**Best E_A (µWh cm^{-2})**	**Best P_A (µW cm^{-2})**	**Best E_L (µW cm^{-1})**	**Best P_L (µW cm^{-1})**	**Best E_G (Wh kg^{-1})**	**Best P_G (W kg^{-1})**	**Best E_V (mWh cm^{-3})**	**Best P_V (mW cm^{-3})**	**Ref.**
*MXene coated yarn electrode	7,360	1,942	536	342									This work
*rGO/MoS_2 coated yarn electrode	4,756	1278	402	222									This work
MXene yarn//rGO/MoS_2 yarn	658.3	177	52.53	30.8	154.5	8146.7	41.5	2189.7	12.3	650	7.2	380.7	This work
MXene@Ag coated nylon fiber	328	50			7.3	132	1.1						[9]
MnO_2/CNT /Graphene@Ni wire//Ni tube	31	9.93			3.1	1989							[10]
Biscrolled MXene/CNT//biscrolled RuO_2/CNT yarn	554.1	27.8	123.2	203.4	167.9	14786.5	8.4	741			61.6	5428	[11]
MoS_2-rGO/CNT//rGO/CNT fibers				5.2							≈2	≈3000	[12]
rGO/CNT polyester yarn//NiCo BOH polyester yarn	133				78.1	14							[13]
CNTs/MXene-TPU hybrid fiber				8.8							1.16	160	[14]
$Ti_3C_2T_x$ MXene coaxial zinc-ion hybrid fiber	214				42.8	12138							[15]
Stretch-broken carbon fiber yarns	0.43				0.0173	533							[16]
MXene coated wool fibers	284				3.7	210							[17]
MXene based wire type supercapacitors		3.09	4.64				0.21	5.8	0.3	8.75			[18]

*Values were normalized to a single electrode.

References

[1] a) E. Berger, Z.-P. Lv, H.-P. Komsa, *Journal of Materials Chemistry C* **2023**, 11, 1311; b) T. Hu, J. Wang, H. Zhang, Z. Li, M. Hu, X. Wang, *Physical Chemistry Chemical Physics* **2015**, 17, 9997.

[2] S. Adomaviciute-Grabusove, A. Popov, S. Ramanavicius, V. Sablinskas, K. Shevchuk, O. Gogotsi, I. Baginskiy, Y. Gogotsi, A. Ramanavicius, *ACS Nano* **2024**, 18, 13184.

[3] R. Thangappan, S. Kalaiselvam, A. Elayaperumal, R. Jayavel, M. Arivanandhan, R. Karthikeyan, Y. Hayakawa, *Dalton Transactions* **2016**, 45, 2637.

[4] a) G. L. Frey, R. Tenne, M. J. Matthews, M. S. Dresselhaus, G. Dresselhaus, *Physical Review B* **1999**, 60, 2883; b) S. Mignuzzi, A. J. Pollard, N. Bonini, B. Brennan, I. S. Gilmore, M. A. Pimenta, D. Richards, D. Roy, *Physical Review B* **2015**, 91, 195411.

[5] V. Natu, M. Benchakar, C. Canaff, A. Habrioux, S. Célérier, M. W. Barsoum, *Matter* **2021**, 4, 1224.

[6] T. Schultz, N. C. Frey, K. Hantanasirisakul, S. Park, S. J. May, V. B. Shenoy, Y. Gogotsi, N. Koch, *Chemistry of Materials* **2019**, 31, 6590.

[7] N. Kumar, S. Kumar, R. Gusain, N. Manyala, S. Eslava, S. S. Ray, *ACS Applied Energy Materials* **2020**, 3, 9897.

[8] Q. Lin, X. Dong, Y. Wang, N. Zheng, Y. Zhao, W. Xu, T. Ding, *Journal of Materials Science* **2020**, 55, 6637.

[9] M. Hu, Z. Li, G. Li, T. Hu, C. Zhang, X. Wang, *Advanced Materials Technologies* **2017**, 2, 1700143.

[10] Q. Kang, J. Zhao, X. Li, G. Zhu, X. Feng, Y. Ma, W. Huang, J. Liu, *Nano Energy* **2017**, 32, 201.

[11] Z. Wang, S. Qin, S. Seyedin, J. Zhang, J. Wang, A. Levitt, N. Li, C. Haines, R. Ovalle-Robles, W. Lei, Y. Gogotsi, R. H. Baughman, J. M. Razal, *Small* **2018**, 14, 1802225.

[12] G. Sun, X. Zhang, R. Lin, J. Yang, H. Zhang, P. Chen, *Angewandte Chemie International Edition* **2015**, 54, 4651.

[13] M. Liu, Z. Cong, X. Pu, W. Guo, T. Liu, M. Li, Y. Zhang, W. Hu, Z. L. Wang, *Advanced Functional Materials* **2019**, 29, 1806298.

[14] G. Wu, Z. Yang, Z. Zhang, B. Ji, C. Hou, Y. Li, W. Jia, Q. Zhang, H. Wang, *Electrochimica Acta* **2021**, 395, 139141.

[15] B. Shi, L. Li, A. Chen, T.-C. Jen, X. Liu, G. Shen, *Nano-Micro Letters* **2021**, 14, 34.

[16] J. T. Carvalho, I. Cunha, J. Coelho, E. Fortunato, R. Martins, L. Pereira, *ACS Applied Energy Materials* **2022**, 5, 11987.

[17] A. Grube, A. A. Shamsabadi, M. D. Firouzjaei, S. I. G. P. Mohamed, L. Hilger, M. Elliott, K. McKenzie, M. Bavarian, *Nano Trends* **2023**, 3, 100014.

[18] K. Krishnamoorthy, P. Pazhamalai, S. Sahoo, S.-J. Kim, *Journal of Materials Chemistry A* **2017**, 5, 5726.
